# Hybrid B- and T-Cell Immunity Associates With Protection Against Breakthrough Infection After Severe Acute Respiratory Syndrome Coronavirus 2 Vaccination in Avon Longitudinal Study of Parents and Children (ALSPAC) Participants

**DOI:** 10.1093/infdis/jiaf246

**Published:** 2025-05-20

**Authors:** Holly E Baum, Marianna Santopaolo, Ore Francis, Emily J Milodowski, Katrina Entwistle, Elizabeth Oliver, Benjamin Hitchings, Divya Diamond, Amy C Thomas, Ruth E Mitchell, Milla Kibble, Kapil Gupta, Natalie Di Bartolo, Paul Klenerman, Anthony Brown, Begonia Morales-Aza, Jennifer Oliver, Imre Berger, Ash M Toye, Adam Finn, Anu Goenka, Andrew D Davidson, Susan Ring, Lynn Molloy, Melanie Lewcock, Kate Northstone, Firona Roth, Nicholas J Timpson, Linda Wooldridge, Alice Halliday, Laura Rivino

**Affiliations:** School of Cellular and Molecular Medicine, Faculty of Health and Life Sciences, University of Bristol, Bristol, United Kingdom; Bristol Vaccine Centre, University of Bristol, Bristol, United Kingdom; School of Cellular and Molecular Medicine, Faculty of Health and Life Sciences, University of Bristol, Bristol, United Kingdom; School of Cellular and Molecular Medicine, Faculty of Health and Life Sciences, University of Bristol, Bristol, United Kingdom; Bristol Veterinary School, Faculty of Health and Life Sciences, University of Bristol, Bristol, United Kingdom; Bristol Veterinary School, Faculty of Health and Life Sciences, University of Bristol, Bristol, United Kingdom; Bristol Veterinary School, Faculty of Health and Life Sciences, University of Bristol, Bristol, United Kingdom; School of Cellular and Molecular Medicine, Faculty of Health and Life Sciences, University of Bristol, Bristol, United Kingdom; Bristol Vaccine Centre, University of Bristol, Bristol, United Kingdom; School of Cellular and Molecular Medicine, Faculty of Health and Life Sciences, University of Bristol, Bristol, United Kingdom; Bristol Vaccine Centre, University of Bristol, Bristol, United Kingdom; School of Cellular and Molecular Medicine, Faculty of Health and Life Sciences, University of Bristol, Bristol, United Kingdom; Population Health Sciences, Bristol Medical School, University of Bristol, Bristol, United Kingdom; Population Health Sciences, Bristol Medical School, University of Bristol, Bristol, United Kingdom; Population Health Sciences, Bristol Medical School, University of Bristol, Bristol, United Kingdom; Department of Applied Mathematics and Theoretical Physics, University of Cambridge, Cambridge, United Kingdom; Department of Twin Research and Genetic Epidemiology, King's College London, London, United Kingdom; School of Biochemistry, Faculty of Health and Life Sciences, University of Bristol, Bristol, United Kingdom; School of Biochemistry, Faculty of Health and Life Sciences, University of Bristol, Bristol, United Kingdom; Division of Structural Biology, Nuffield Department of Medicine, University of Oxford, Oxford, United Kingdom; Peter Medawar Building for Pathogen Research, University of Oxford, Oxford, United Kingdom; School of Cellular and Molecular Medicine, Faculty of Health and Life Sciences, University of Bristol, Bristol, United Kingdom; Bristol Vaccine Centre, University of Bristol, Bristol, United Kingdom; Bristol Vaccine Centre, University of Bristol, Bristol, United Kingdom; Population Health Sciences, Bristol Medical School, University of Bristol, Bristol, United Kingdom; School of Biochemistry, Faculty of Health and Life Sciences, University of Bristol, Bristol, United Kingdom; School of Biochemistry, Faculty of Health and Life Sciences, University of Bristol, Bristol, United Kingdom; School of Cellular and Molecular Medicine, Faculty of Health and Life Sciences, University of Bristol, Bristol, United Kingdom; Bristol Vaccine Centre, University of Bristol, Bristol, United Kingdom; Population Health Sciences, Bristol Medical School, University of Bristol, Bristol, United Kingdom; Department of Paediatric Immunology and Infectious Diseases, Bristol Royal Hospital for Children, Bristol, United Kingdom; School of Cellular and Molecular Medicine, Faculty of Health and Life Sciences, University of Bristol, Bristol, United Kingdom; Bristol Vaccine Centre, University of Bristol, Bristol, United Kingdom; Population Health Sciences, Bristol Medical School, University of Bristol, Bristol, United Kingdom; Department of Paediatric Immunology and Infectious Diseases, Bristol Royal Hospital for Children, Bristol, United Kingdom; School of Cellular and Molecular Medicine, Faculty of Health and Life Sciences, University of Bristol, Bristol, United Kingdom; Population Health Sciences, Bristol Medical School, University of Bristol, Bristol, United Kingdom; Medical Research Council Integrative Epidemiology Unit, University of Bristol, Bristol, United Kingdom; Population Health Sciences, Bristol Medical School, University of Bristol, Bristol, United Kingdom; Population Health Sciences, Bristol Medical School, University of Bristol, Bristol, United Kingdom; Population Health Sciences, Bristol Medical School, University of Bristol, Bristol, United Kingdom; Population Health Sciences, Bristol Medical School, University of Bristol, Bristol, United Kingdom; Population Health Sciences, Bristol Medical School, University of Bristol, Bristol, United Kingdom; Medical Research Council Integrative Epidemiology Unit, University of Bristol, Bristol, United Kingdom; Bristol Veterinary School, Faculty of Health and Life Sciences, University of Bristol, Bristol, United Kingdom; School of Cellular and Molecular Medicine, Faculty of Health and Life Sciences, University of Bristol, Bristol, United Kingdom; Bristol Vaccine Centre, University of Bristol, Bristol, United Kingdom; School of Cellular and Molecular Medicine, Faculty of Health and Life Sciences, University of Bristol, Bristol, United Kingdom

**Keywords:** ALSPAC, SARS-CoV-2, vaccination, hybrid immunity, breakthrough infection

## Abstract

**Background:**

Immunological memory to vaccination and viral infection involves the coordinated action of B and T cells; thus, integrated analysis of these 2 components is critical for understanding their respective contributions to protection against breakthrough infections (BIs) after vaccination.

**Methods:**

We investigated cellular and humoral immune responses to severe acute respiratory syndrome coronavirus 2 (SARS-CoV-2) infection and/or vaccination in 300 adult participants from the Avon Longitudinal Study of Parents and Children (ALSPAC). Participants were grouped by those with (cases) and without (controls) a history of SARS-CoV-2 infection. To provide a quantitative correlate for protection against BI in the 8-month period after the study, Youden index thresholds were calculated for all immune measures analyzed.

**Results:**

The magnitude of antibody and T-cell responses following the second vaccine dose was associated with protection against BI in participants with a history of SARS-CoV-2 infection (cases), but not in infection-naive controls. Over 8 months of follow-up, 2 threshold combinations provided the best performance for protection against BI in cases: (*i*) anti-spike immunoglobulin G (IgG) (≥666.4 binding antibody units [BAU]/mL) combined with anti-nucleocapsid pan-immunoglobulin (pan-Ig) (≥0.1332 BAU/mL) and (*ii*) spike 1–specific T cells (≥195.6 spot-forming units/10^6^ peripheral blood mononuclear cells) combined with anti-N pan-Ig (≥0.1332 BAU/mL). Both combinations offered 100% specificity for detecting cases without BI, with sensitivities of 83.3% and 72.2%, respectively.

**Conclusions:**

Collectively, these results suggest that hybrid B- and T-cell immunity offers superior protection from BI after coronavirus disease 2019 (COVID-19) vaccination, and this finding has implications for designing next-generation COVID-19 vaccines that are capable of eliciting immunity to a broader repertoire of SARS-CoV-2 proteins.

## Teaser

The magnitude of antibody and T-cell responses to COVID-19 vaccination associate with protection against breakthrough infections in participants with previous SARS-CoV-2 infection. Combining thresholds for anti-nucleocapsid pan-immunoglobulin, and anti-spike IgG or spike 1–specific T cells, identifies protected individuals with 100% specificity.

The coronavirus disease 2019 (COVID-19) pandemic caused substantive medical and socioeconomic burdens that continue to affect the global population [1, 2]. Infection with severe acute respiratory syndrome coronavirus 2 (SARS-CoV-2) presents as a broad spectrum of clinical manifestations from asymptomatic or mild infections, through to severe illness, and associated mortality [3]. The rapid deployment of COVID-19 vaccines proved instrumental in reducing SARS-CoV-2 infections and subsequent hospitalizations and deaths; however, vaccine effectiveness wanes over time [4]. Breakthrough infections (BIs) are well documented and influenced not only by waning immunity, but also the evolution of novel viral variants [5, 6].

A broad repertoire of antibody and cellular responses are elicited by SARS-CoV-2 infection [7, 8] and COVID-19 vaccination [9, 10]. Evidence for anti-spike (S) immunoglobulin G (IgG) and neutralizing antibody (nAb) titers as correlates of protection against SARS-CoV-2 infection and severe disease have been demonstrated in vaccine trials [11, 12] and studies of BIs [6]. Additionally, systemic and mucosal immunoglobulin A (IgA) have been implicated in protection against infection [13]. SARS-CoV-2–specific T-cell responses are more durable than antibodies [14] and may therefore be important for protection longer-term, and against novel variants [15] that can evade antibody-mediated immunity [16]. Furthermore, cross-reactive T cells can enhance infection- and vaccine-mediated responses [17] and induce abortive infections in highly exposed individuals [18].

Antiviral immune memory responses are determined by the coordinated action of antibodies and T cells targeting the virus; therefore, an integrated analysis is critical for understanding their respective contributions to protection. Here, we investigate cellular and humoral responses to SARS-CoV-2 following infection and/or vaccination in participants from the Avon Longitudinal Study of Parents and Children (ALSPAC). We further address the role of antibodies and T cells in protecting against BI in participants with hybrid immunity, gained through a combination of SARS-CoV-2 infection and vaccination, compared to those with vaccine-induced immunity alone.

## METHODS

In addition to the referenced methods below, full details are provided in the Supplementary Materials.

### ALSPAC

ALSPAC is a birth cohort study that invited pregnant women resident in Avon, United Kingdom (UK), with expected dates of delivery between 1 April 1991 and 31 December 1992 to participate [19–22]. The cohort comprises 3 generations: the original pregnant women and biological fathers/partners (G0), the index children (G1), and the offspring of the index children (G2).

### Study Design

In October 2020, ALSPAC undertook 5200 serological SARS-CoV-2 spike–specific IgG lateral flow test (LFTs) on G0/G1 participants [23]. Those with evidence of a previous SARS-CoV-2 infection based on a positive LFT, and/or positive SARS-CoV-2 polymerase chain reaction (PCR) result from linked UK Health Security Agency data, were invited to participate (n = 124). Two control groups with negative serological results, and no documented positive PCR test, were recruited alongside. The first control group (n = 93) was age, sex, and symptom (anosmia) matched to those with a history of SARS-CoV-2 infection. The second control group (n = 103) did not report anosmia. The full recruitment methodology is described in Mitchell et al [24]. In brief, participants attended clinics in December 2020 (clinic 1), March 2021 (clinic 2), and June 2021 (clinic 3) and provided blood and saliva samples. Additional participants were recruited at clinic 2 and clinic 3 to maintain numbers and account for withdrawals. Health/lifestyle information was gathered through online questionnaires. Control groups have been combined for these analyses as no significant differences in baseline antibody/T-cell measures were detected between the 2 original groups [24].

### Ethics

Ethical approval for the study was obtained from the ALSPAC Ethics and Law Committee and the Local Research Ethics Committees (NHS REC 20/HRA/4854). Consent for biological samples was collected in accordance with the Human Tissue Act (2004). Informed consent for using data collected via questionnaires and clinics was obtained from participants following the recommendations of the ALSPAC Ethics and Law Committee.

### Sample Processing

Whole blood was processed to peripheral blood mononuclear cells (PBMCs) and serum, as previously described [24]. Neat saliva was centrifuged at 13 000*g* for 10 minutes to remove solids.

### Immunological Assays

SARS-CoV-2–specific anti-spike (S) and anti-nucleocapsid (N) antibody levels were measured by enzyme-linked immunosorbent assay (ELISA) in serum [25] and saliva [26], as previously described. Serum nAb titers were measured using a pseudotype assay on samples from the case group with normalized anti-S pan-immunoglobulin (pan-Ig) ELISA results >0.5, as previously described [25].

Interferon gamma (IFN-γ) enzyme-linked immunosorbent spot (ELISpot) assays were performed as documented [24], using peptides spanning SARS-CoV-2 S1, S2, membrane (M), N, envelope (E), open reading frame (ORF) 1 (NSP1 + 2, NSP3A, NSP3B, NSP3C, NSP4, NSP5 + 6, NSP7-11, NSP12A, NSP12B, NSP13, NSP14, NSP15 + 16), ORF3, ORF6, ORF7, and ORF8. Intracellular cytokine staining (ICS) using peptides spanning S1/S2, M, N, and NSP3B was performed for participants from the case group with detectable baseline IFN-γ ELISpot T-cell responses against ≥1 peptide pool (Supplementary Table 4) [27].

### Data Analysis

Statistical analyses were performed using R Studio (v4.3.0) and GraphPad Prism (v10.01) software. Data were removed if no corresponding information on COVID-19 vaccination status was available. Where participants provided samples at >1 clinic after a specified vaccine dose (including prevaccination), only data from the earliest timepoint were analyzed.

## RESULTS

### Study Design and Recruitment

**Figure 1.**
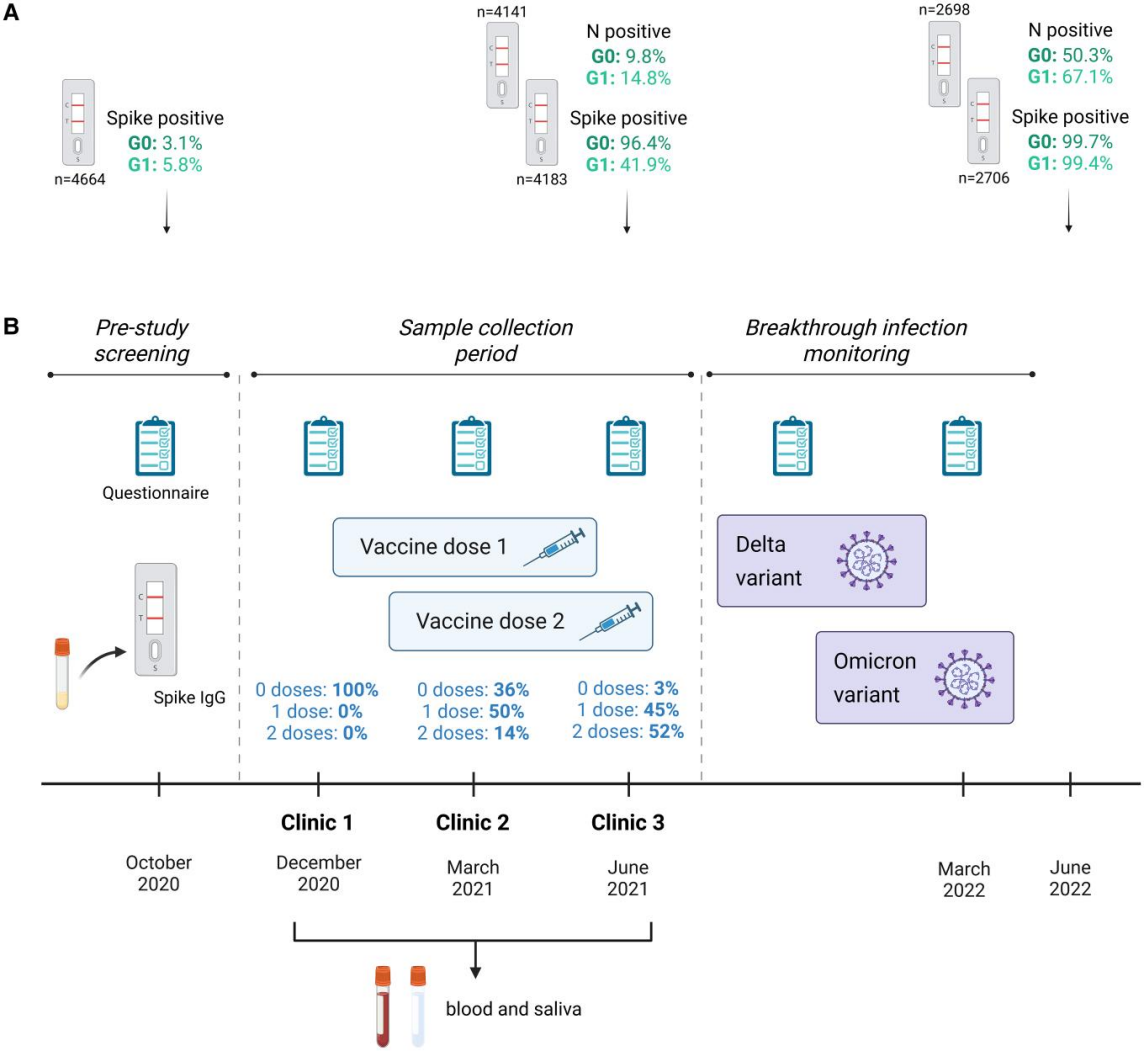
Study design. *A*, Participants from the Avon Longitudinal Study of Parents and Children (ALSPAC) were screened for severe acute respiratory syndrome coronavirus 2 (SARS-CoV-2)–specific anti-spike (S) immunoglobulin G (IgG) in October 2020, prior to the coronavirus disease 2019 (COVID-19) vaccine rollout. Both the G0 (age 48–70 y) and G1 (age 29–30 y) generations were eligible for screening. Further sampling was conducted in June 2021 and June 2022 for both anti-S and anti-nucleocapsid (N) IgG. Numbers indicate the total valid tests, with the percentages indicating the positivity rates. *B*, Of the 4819 participants with valid lateral flow test (LFT) results in October 2020, 377 were recruited to this study. Participants attended 1 or more clinics from December 2020 to June 2021, providing biological samples and completing health questionnaires. During this period, participants became eligible to receive COVID-19 vaccines via the UK national vaccination program (text above clinic timepoints indicates percentage vaccinated at each clinic). Following the sampling period, participants continued to complete online questionnaires detailing their LFT and polymerase chain reaction–confirmed SARS-CoV-2 infections. Created in BioRender (A. Halliday, 2025; https://BioRender.com/i7wpnfr).

This study was open to ALSPAC participants of the G0 (48–70 years) and G1 (29–30 years) generations. Three hundred seventy-seven participants enrolled and attended clinics in December 2020, March 2021, and June 2021 (Figure 1). Participants were 59.2% female, 97.8% white, and 58.5% generation G1 (Supplementary Table 1). Participants were selected from 4819 individuals with valid SARS-CoV-2 anti-S IgG LFT results. Positivity rates, indicative of historic SARS-CoV-2 infections, were 3.1% and 5.8% in the G0 and G1 generations, respectively (Figure 1) [24].

**Figure 2.**
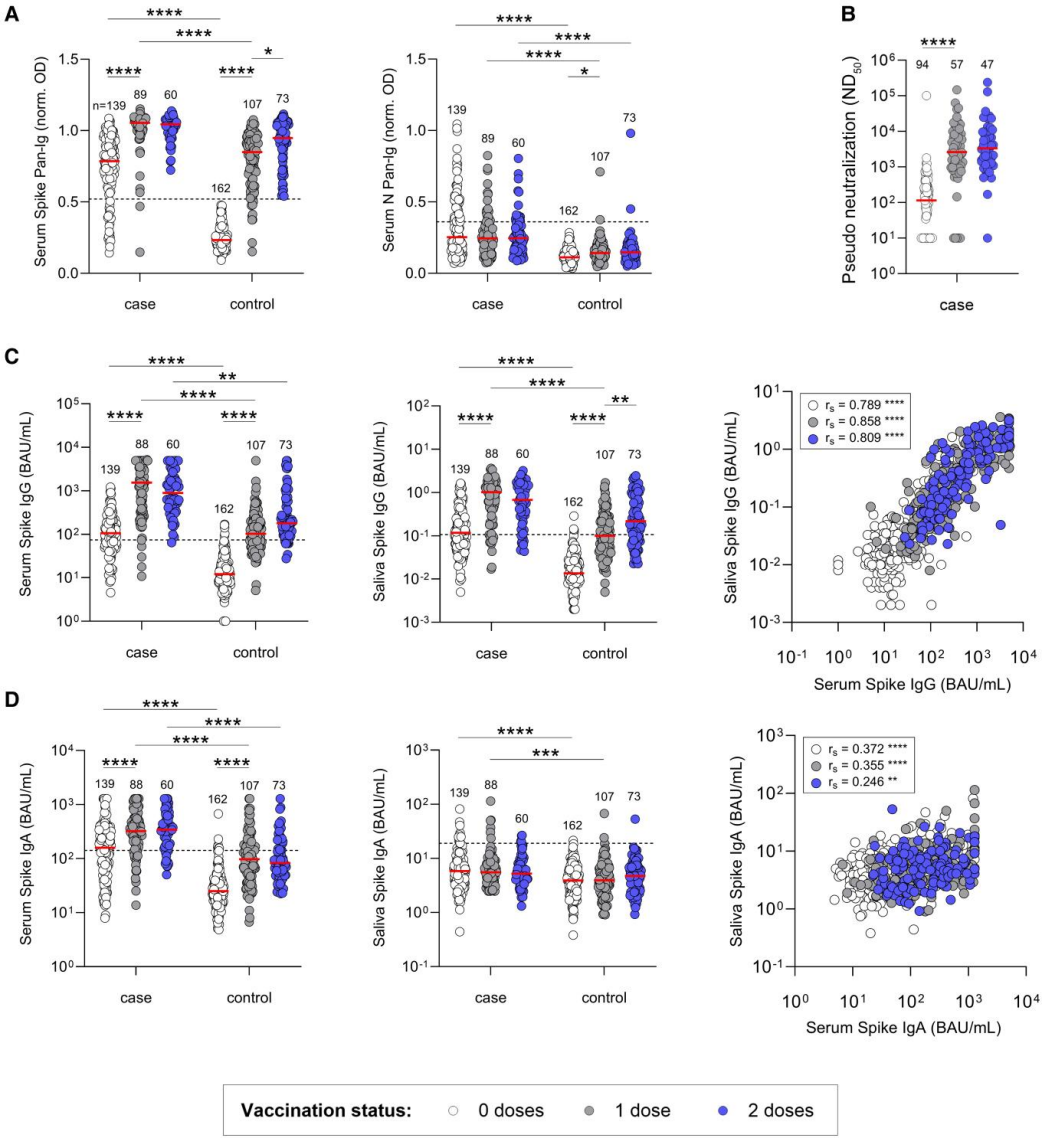
Antibody responses to coronavirus disease 2019 vaccination in severe acute respiratory syndrome coronavirus 2 (SARS-CoV-2)–naive and previously infected individuals. *A*, Participants were classified as cases based on a previous polymerase chain reaction–confirmed SARS-CoV-2 infection and/or positivity on serum anti-spike and/or anti-nucleocapsid pan-immunoglobulin enzyme-linked immunosorbent assays (ELISAs). *B*, Serum pseudoneutralizing antibody titers. *C* and *D*, Anti-spike immunoglobulin G and immunoglobulin A in serum and saliva, measured by ELISA. Correlation coefficients were calculated using Spearman rank test (r_s_). Central bars indicate median responses. Unpaired comparisons were performed using Kruskal-Wallis test with Dunn correction for multiple comparisons. Within each of the case and control groups, responses were compared between 0 and 1, and 1 and 2 vaccine doses. Responses between groups were compared after each dose. Pairwise correlations were assessed with Spearman rank-order correlation (r_s_). Correlation coefficients were interpreted as weak (r_s_ = 0.20–0.39), moderate (r_s_ = 0.40–0.59), strong (r_s_ = 0.60–0.79), or very strong (r_s_ = 0.80–1.00). Statistics are only displayed for comparisons where *P ≤ .05, **P ≤ .01, ***P ≤ .001, and ****P ≤ .0001. Abbreviations: BAU, binding antibody units; IgA, immunoglobulin A; IgG, immunoglobulin G; N, nucleocapsid; ND_50_, 50% neutralising dilution; OD, optical density; pan-Ig, pan-immunoglobulin.

To validate the LFT results, serum samples from clinic 1 were screened for SARS-CoV-2–specific anti-S and anti-N antibodies by ELISA. Participants were defined as “cases” if they had a previous PCR-confirmed SARS-CoV-2 infection and/or were positive on the anti-N or anti-S ELISAs (Figure 2*A*). Seronegative individuals with no COVID-19 history are herein referred to as controls. Clinic 1 samples were taken before participants were vaccinated during the UK COVID-19 vaccination program. Of those who reported their vaccination status, 64.0% received ≥1 dose by clinic 2, and 97.0% by clinic 3 (Figure 1). Data were therefore analyzed relative to the number of vaccinations received (Supplementary Figure 1), rather than chronologically by clinic, as this was deemed the dominant variable influencing immune responses.

### Antibody Responses to COVID-19 Vaccination

Baseline anti-S and anti-N pan-Ig levels were higher in cases versus controls (*P* ≤ .0001; Figure 2*A*). A vaccine-induced increase in anti-S pan-Ig was observed in both groups. Following dose 2, all participants had anti-S pan-Ig levels above the assay positivity threshold. However, the case group retained their baseline advantage with higher median titers. Rates of new SARS-CoV-2 infections between clinics 1 through 3 were low; 4 individuals in the control group reported positive PCR tests. Consistent with this, a small increase in median anti-N pan-Ig was observed after dose 1 (*P* = .0471), which plateaued after dose 2 (Figure 2*A*). This was predominantly driven by ≥2-fold increases in anti-N pan-Ig levels in a small minority of participants (3/95; Supplementary Figure 2). Anti-N pan-Ig levels in the case group remained stable.

Serum and salivary anti-S IgG showed similar kinetics to serum anti-S pan-Ig. Postvaccination anti-S IgG levels in the 2 sample types were very strongly correlated after dose 1 (r_s_ = 0.858, *P* ≤ .0001) and dose 2 (r_s_ = 0.809, *P* ≤ .0001) (Figure 2*C*). In cases, serum pseudo-viral neutralizing antibody (PsVNA) titers increased after dose 1, then stabilized (Figure 2*B*). Anti-S IgG strongly correlated with PsVNA titers after both doses (r_s_ = 0.784, *P* ≤ .0001/r_s_ = .656, *P* ≤ .0001; Supplementary Figure 3).

Prevaccination anti-S IgA in serum and saliva was higher in cases than controls (Figure 2*D*). Serum anti-S IgA increased in both groups after dose 1, while median salivary anti-S IgA levels were not boosted. The 2 measures were therefore only weakly correlated postvaccination (r_s_ = 0.355, *P* ≤ .0001 / r_s_ = .246, *P* = .0045). Messenger RNA (mRNA) vaccine recipients had higher median serum anti-S IgG and IgA levels than recipients of the adenoviral vector–based ChAdOx1 vaccine (Supplementary Figure 4).

### T-Cell Responses to COVID-19 Vaccination

**Figure 3.**
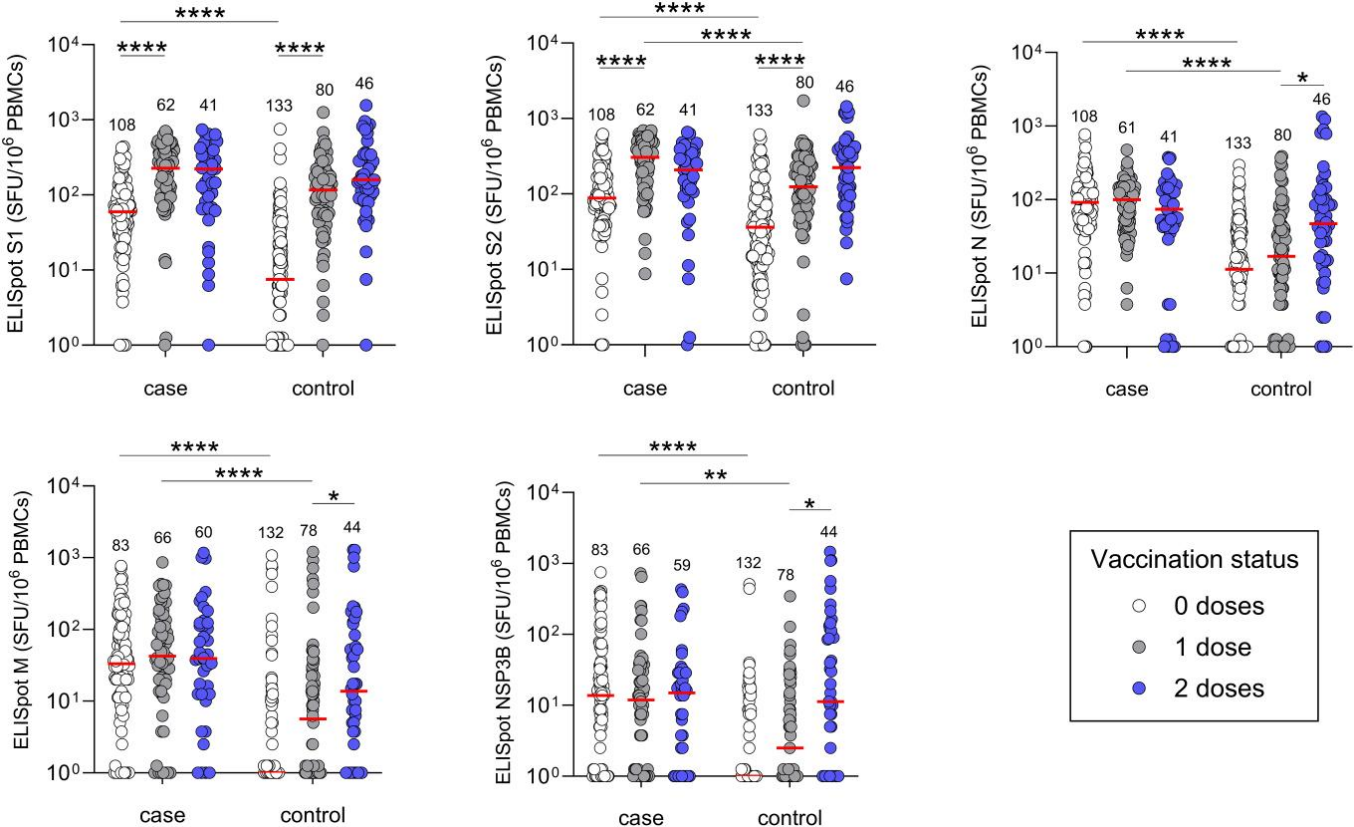
T-cell responses to coronavirus disease 2019 vaccination in severe acute respiratory syndrome coronavirus 2 (SARS-CoV-2)–naive and previously infected individuals. Magnitude of SARS-CoV-2–specific T-cell responses against spike 1, spike 2, nucleocapsid, membrane, and NSP3B peptide pools measured by enzyme-linked immunosorbent spot assay. Participants were classified as cases based on a previous polymerase chain reaction–confirmed SARS-CoV-2 infection and/or positivity on serum anti-spike and/or anti-nucleocapsid pan-immunoglobulin enzyme-linked immunosorbent assays (see Figure 2). Central bars indicate median responses. Unpaired comparisons were performed using Kruskal-Wallis test with Dunn correction for multiple comparisons. Within each of the case and control groups, responses were compared between 0 and 1, and 1 and 2 vaccine doses. Responses between groups were compared after each dose. Statistics are only displayed for comparisons where *P ≤ .05, **P ≤ .01, and ****P ≤ .0001. To facilitate the presentation of data that included zero counts on log scales, zero counts were plotted as 1 for visualization purposes only; all statistical analyses were performed on the raw data values. Abbreviations: ELISpot, enzyme-linked immunosorbent spot assay; N, nucleocapsid; PBMC, peripheral blood mononuclear cell; SFU, spot-forming unit.

T-cell responses targeting SARS-CoV-2 proteins were measured by IFN-γ ELISpot (Supplementary Figure 5*A* and 5*B*). Baseline S-specific responses were lower in controls versus cases (*P* ≤ .0001; Figure 3). In both groups, S-specific T cells increased after dose 1 and then stabilized. In cases, responses to N, M, and NSP3B were not boosted by vaccination, while those to NSP1 + 2 did increase. In contrast, increased responses to M (*P* ≤ .0001), N (*P* = .0175), and NSP3B (*P* = .0091) were observed in control participants after dose 2 versus baseline (Figure 3). T-cell responses to NSP3-16, ORF3, ORF7, and ORF8 remained stable following vaccination (Supplementary Figure 5*A* and 5*B*).

### Magnitude and Quality of T-Cell Responses to SARS-CoV-2 Infection

**Figure 4.**
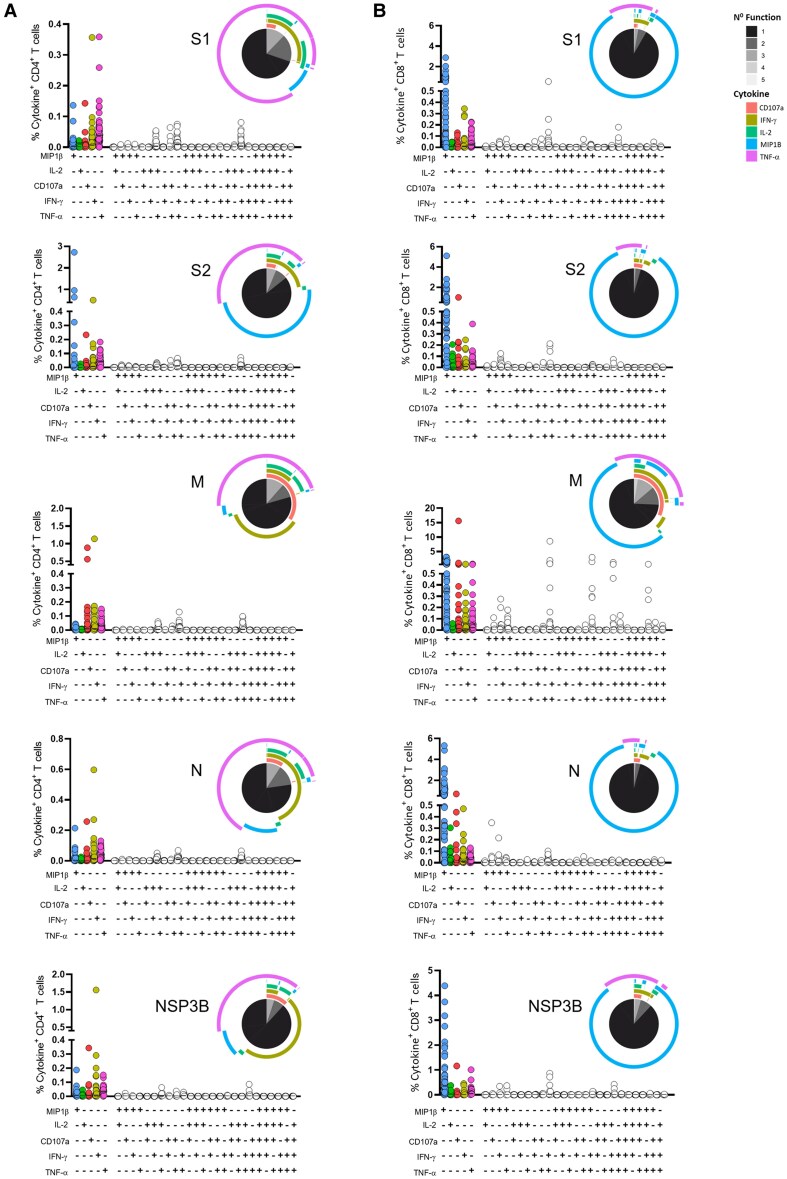
Severe acute respiratory syndrome coronavirus 2 (SARS-CoV-2)–specific CD4^+^ and CD8^+^ T-cell responses in previously infected individuals. Graphs show the percentage of CD4^+^ (*A*) and CD8^+^ (*B*) T cells producing the indicated cytokine or combination of cytokines after a brief stimulation with spike 1, spike 2, membrane, nucleocapsid, or NSP3B peptide pools, assessed by intracellular cytokine staining and flow cytometry. Pie charts indicate the proportion of T cells, within total cytokine^+^ T cells, producing each cytokine, and that display 1 or more functions. Statistics from monofunctional SARS-CoV-2–specific CD4^+^ and CD8^+^ T cells in previously infected individuals were calculated using a Kruskal-Wallis test with false discovery rate method of Benjamini and Hochberg correction for multiple comparisons. *P* values are reported in Supplementary Table 2. To facilitate the presentation of data that included zero counts on log scales, zero counts were plotted as 1 for visualization purposes only; all statistical analyses were performed on the raw data values. Abbreviations: IFN-γ, interferon gamma; IL-2, interleukin 2; M, membrane; MIP-1β, macrophage inflammatory protein 1β; N, nucleocapsid; S, spike; TNF-α, tumor necrosis factor alpha.

The magnitude and functional phenotypes of prevaccination T-cell responses targeting S1/2, N, M, and NSP3B were analyzed in cases. Production of IFN-γ, tumor necrosis factor alpha (TNF-α), macrophage inflammatory protein 1β (MIP1β), interleukin 2 (IL-2), and degranulation (CD107a) by SARS-CoV-2–specific CD4^+^/CD8^+^ T cells was measured by ICS (Supplementary Figure 6). Responses comprised monofunctional and polyfunctional T cells producing 1 or >1 effector functions, respectively, with monofunctional T cells dominating the CD4^+^ and CD8^+^ T-cell response (Figure 4 and Supplementary Table 2). The frequencies of monofunctional CD4^+^ and CD8^+^ T cells producing each cytokine were comparable across proteins (Supplementary Figure 7*A* and 7*B*). In contrast, CD107a was expressed at higher levels by CD4^+^ and CD8^+^ T cells targeting M, compared to the other proteins (Supplementary Figure 7*A* and 7*B*). Polyfunctional CD4^+^ and CD8^+^ T cells were mainly double- or triple-cytokine–producing, including IFN-γ^+^/TNF-α^+^, IL-2^+^/TNF-α^+^, and IFN-γ^+^/IL-2^+^/TNF-α^+^ for CD4^+^ T cells (Figure 4*A*) and IFN-γ^+^/TNF-α^+^, CD107a^+^/TNF-α^+^, MIP1β^+^/TNF-α^+^, MIP1β^+^/IFN-γ^+^, and MIP1β^+^/IFN-γ^+^/TNF-α^+^ for CD8^+^ T cells (Figure 4*B*). Polyfunctional CD4^+^ and CD8^+^ T cells targeting NSP3B displayed higher polyfunctionality than those targeting S2, M and N, or S1/S2 (Figure 4*A* and 4*B*; Supplementary Figure 8*A* and 8*B*).

#### Association Between Postvaccination Immune Responses and Protection Against SARS-CoV-2 BI

We next investigated the relationship between the magnitude of antibody and T-cell responses after the second vaccination, and protection against SARS-CoV-2 BI. Case and control groups were subdivided based on self-reported PCR/LFT-confirmed SARS-CoV-2 infections in the 8 months following the sample collection period (July 2021–March 2022; Figure 1). The case group was updated to include 2 individuals with PCR-confirmed SARS-CoV-2 infection between clinics 1–3. BI rates were higher in controls (42.4%) than cases (30.8%). Of those who reported BIs, the median time since second vaccination, and the proportion who received a third vaccination before infection, were comparable between cases and controls (Supplementary Table 3). Self-reported BIs were consistent with increased rates of LFT anti-N IgG positivity in the wider ALSPAC cohort from May 2021 (G1 = 14.8%; G0 = 9.8%) to May 2022 (G1 = 67.1%; G0 = 50.3%) (Figure 1).

**Figure 5.**
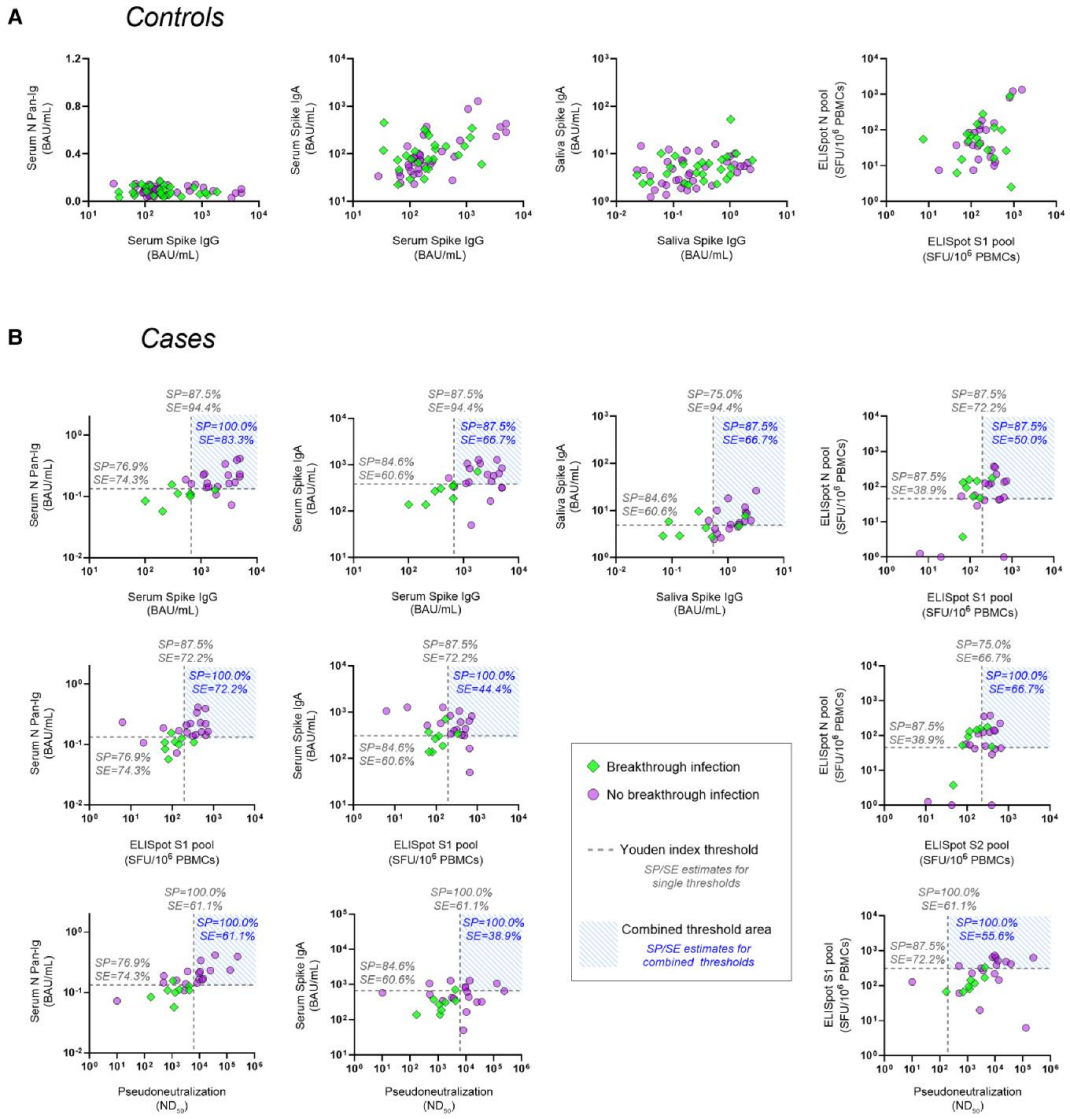
Association between immune responses to prior severe acute respiratory syndrome coronavirus 2 (SARS-CoV-2) infection and coronavirus disease 2019 vaccination, and susceptibility to SARS-CoV-2 breakthrough infection (BI). Correlation of post–second vaccination antibody and T-cell responses in participants who self-reported a SARS-CoV-2 infection in the subsequent 8 months (BI) and those who did not (no BI). *A*, SARS-CoV-2-naive individuals (with BI, n = 14; no BI, n = 19). *B*, Previously SARS-CoV-2–infected individuals (BI, n = 8; no BI, n = 18). See Supplementary Figure 10 for derivation of Youden index thresholds. Abbreviations: BAU, binding antibody units; ELISpot, enzyme-linked immunosorbent spot assay; IgA, immunoglobulin A; N, nucleocapsid; ND_50_, 50% neutralising dilution; pan-Ig, pan-immunoglobulin; PBMC, peripheral blood mononuclear cell; S, spike; SE, sensitivity; SFU, spot-forming unit; SP, specificity.

In controls, there was no strong evidence of differences in the magnitude of immune variables measured between those who went on to experience a BI compared with those who did not (Supplementary Figure 9*A* and Figure 5*A*). Conversely, cases showed clustering by subsequent infection status (Figure 5*B*), which was supported by hierarchical clustering analysis (Supplementary Figure 11). Higher serum/saliva anti-S IgG, PsVNA, serum anti-S IgA, and S1-specific T-cell responses were observed in those without BIs (Supplementary Figure 9*B*). Similarly, high anti-N pan-Ig titers were associated with a decreased likelihood of reinfection. The lack of vaccine-induced salivary IgA in this cohort was reflected in the poor performance of this measure as a discriminator of BI susceptibility.

To compare the effectiveness of individual markers for discriminating between cases with or without BIs, thresholds were calculated using the Youden index method to balance sensitivity and specificity (Supplementary Figure 10). Anti-S IgG ≥666.4 BAU/mL in serum (specificity [SP] = 87.5%), or ≥0.547 BAU/mL in saliva (SP = 75.0%), provided 94.4% sensitivity for identifying participants without BIs (Figure 5). Specificity improved to 100.0% by combining serum (or saliva) S-specific IgG and N-specific pan-Ig thresholds (SP = 100%, sensitivity [SE] = 83.3%). Combining S-specific serum IgG with IgA in serum (or saliva), or with T-cell responses, did not improve discrimination. PsVNA ≥6165 units provided a highly specific threshold (SP = 100%) for identifying protected individuals, albeit with lower sensitivity (SE = 61.1%) than S-specific IgG (SE = 94.4%).

The magnitude of T-cell responses to S1 (SP = 87.5%, SE = 72.2%) provided a more specific and sensitive marker of infection susceptibility than those to S2 (SP = 75.0%, SE = 66.67%) or N (SP = 87.5%, SE = 38.9%). Combining thresholds for S1 and N did not improve performance compared to S1 alone. Conversely, combining T-cell S1 with N-specific pan-Ig improved specificity with no loss of sensitivity (SP = 100%, SE = 72.2%). Performances of all threshold combinations are detailed in Supplementary Figure 12.

#### Association Between ICS Responses to Prevaccination SARS-CoV-2 Infection and BI

**Figure 6.**
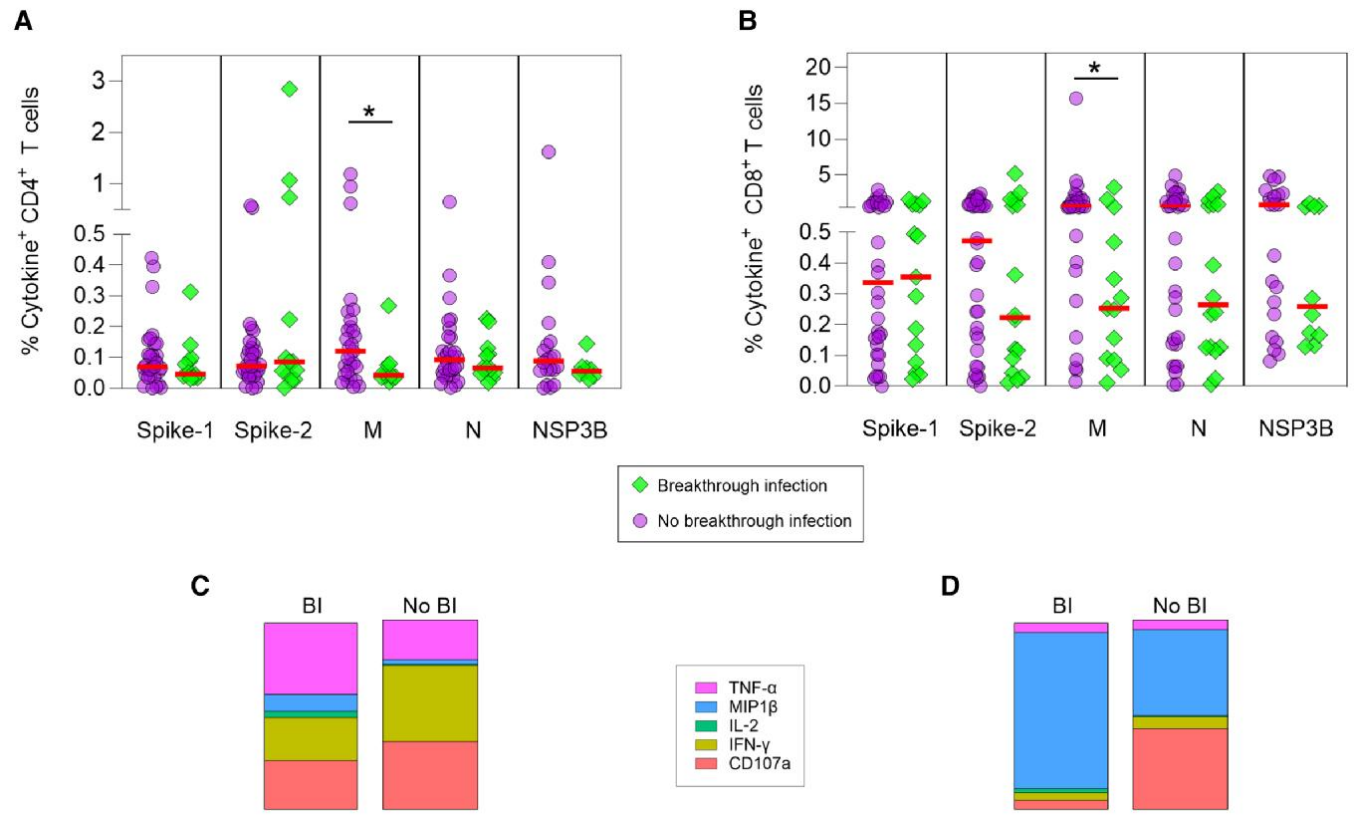
Association between prevaccination severe acute respiratory syndrome coronavirus 2 (SARS-CoV-2)–specific T-cell intracellular cytokine responses and susceptibility to breakthrough infection. Correlation between SARS-CoV-2–specific T-cell intracellular cytokine responses in participants with a history of SARS-CoV-2 infection prior to coronavirus disease 2019 vaccination (cases) and postvaccination breakthrough infections. *A* and *B*, Magnitude of baseline (prevaccination) single cytokine/CD107a-producing (monofunctional) SARS-CoV-2–specific CD4^+^ (*A*; n = 47) and CD8^+^ (*B*; n = 51) T cells specific for the indicated SARS-CoV-2 proteins among cases. Central bars represent median responses. Statistics calculated by *t* test (Mann-Whitney). *C* and *D*, Proportions of single cytokine/CD107a-producing CD4^+^ (*C*; n = 40) and CD8^+^ (*D*; n = 43) T cells within the respective membrane-specific T-cell population. Statistics are only displayed for comparisons where *P ≤ .05. Abbreviations: BI, breakthrough infection; IFN-γ, interferon gamma; IL-2, interleukin 2; M, membrane; MIP-1β, macrophage inflammatory protein 1β; N, nucleocapsid; TNF-α, tumor necrosis factor alpha.

To determine whether specific features of the T-cell response to SARS-CoV-2 infections were also a factor in determining risk of BI, prevaccination ICS responses were compared in cases with or without BIs. Monofunctional CD4^+^ and CD8^+^ T-cell responses were included in these analyses as these were the predominant responses observed. There was no meaningful difference between the magnitude of S-, N-, or NS3PB-specific T-cell responses in the 2 groups (Figure 6*A* and 6*B* and Supplementary Figure 8*C* and 8*D*). In contrast, participants without BIs displayed higher magnitudes of M-specific CD4^+^ and CD8^+^ T cells, and these were skewed toward production of IFN-γ^+^ and CD107a (IFN-γ^+^: 40.0%; CD107a: 35.7%; Figure 6*C*), and CD107a and MIP1β (CD107a: 42.4%; MIP1β: 45.0%; Figure 6*D*), respectively. However, incorporating ICS measures did not improve the predictive ability of thresholds generated using antibody and/or T-cell data alone.

## DISCUSSION

Humoral and cellular immune responses to COVID-19 vaccination were measured in ALSPAC participants with or without prior SARS-CoV-2 infection. In those with hybrid immunity, we demonstrate a correlation between the magnitude of responses following the second vaccination and protection against BI. Combining serum S-specific IgG (≥666.4 BAU/mL) and N-specific pan-Ig (≥0.1332 BAU/mL) thresholds identified those without reported BIs with 100% specificity and 83% sensitivity. Our results suggest that hybrid immunity to SARS-CoV-2 remains effective in protection from reinfection at >15 months postinfection and >8 months post–second dose vaccination. The reduced association in control participants alludes to the importance of the greater quality and breadth of immune responses SARS-CoV-2 infection elicits compared to vaccination alone.

COVID-19 vaccination elicited robust serum anti-S IgG and IgA production in all participants, with cases retaining their baseline immunological advantage as observed in other studies [28]. mRNA vaccination resulted in higher S-specific IgG and IgA compared to the viral vector ChAdOx1, as previously reported [29]. However, neither vaccine type induced salivary IgA, even in participants with a history of COVID-19 where vaccination has been suggested to boost infection-primed responses [13, 30]. Reducing or eliminating transmission with vaccines capable of eliciting mucosal immunity therefore remains a priority, and several candidates are currently in development [31].

Consistent with recent reports, COVID-19 vaccines induced S-specific T-cell responses in all participants [10]. An increase in the median frequency of T-cell responses to M, N, and NSP3B was observed in controls. This was predominantly driven by large increases to all antigens in a small number of participants, suggestive of undetected SARS-CoV-2 infections in these individuals during the study. No parallel increases in serum anti-N pan-Ig or saliva anti-S IgA were observed; however, these markers may have waned before sampling [24].

Long-term protection against SARS-CoV-2 BI relies upon the durability of vaccine-induced responses. Antibodies in particular wane rapidly in the 6 months following the primary and subsequent vaccine doses [32]. This declining immunity, particularly in the context of highly transmissible SARS-CoV-2 variants with reduced neutralizing sensitivity, increases BI rates [4, 5]. Anti-S IgG and nAb levels negatively correlate with SARS-CoV-2 infection risk and severe disease [12, 33]. In controls, we observed no difference in the magnitude of responses between those with and without BIs, and protective thresholds therefore could not be defined. However, anti-S IgG ≥666.4 BAU/mL was associated with reduced BIs in those with a prior SARS-CoV-2 infection. This is consistent with the large Israeli COVID-19 Family Study (ICoFS), which proposed S-specific IgG >500 BAU/mL and nAb titers >1024 as protective thresholds against SARS-CoV-2-Delta infection [34]. ICoFS recorded baseline measurements shortly before infection, and their lower threshold value aligns with our use of post–second vaccination titers recorded months earlier. Serum anti-S IgA was associated with reduced BIs in our cohort, and alongside mucosal IgA has previously been linked to protection against infection independent of IgG [13]. BIs experienced shortly after primary vaccination correlated with lower vaccine-induced IgA [13], and in those who experienced infections, serum IgA levels inversely correlated with symptom duration [35]. Increased S-specific T cells also correlated with reduced BI, consistent with previous reports for both CD4^+^ and CD8^+^ responses [36]. Furthermore, the combination of high nAbs and S-specific IFN-γ responses is associated with protection against BI [37].

Growing evidence indicates that hybrid immunity affords more robust and sustained protection against BIs than vaccination alone [32, 38–40]. Consistent with this, BI rates were lower in our cases (30.8%) than controls (42.4%). Humoral and cellular immune responses are reported to be qualitatively superior in those infected with SARS-CoV-2 before vaccination [39, 40], and infection-derived immunity may be critical in the context of BIs with novel variants [41]. Only SARS-CoV-2 infection induces a significant and durable systemic IgA response [8], while COVID-19 vaccines are poor inducers of mucosal IgA [42]. Hybrid immunity also increases the magnitude and breadth of neutralizing activity against divergent variants of concern [43]. Additionally, vaccine-induced Fc-receptor binding antibodies, important for the control and clearance of infection, are more abundant in previously infected individuals and remain elevated postvaccination [39].

Functional and phenotypic properties of SARS-CoV-2 adaptive immunity reportedly differ in response to SARS-CoV-2 infection and vaccination [44]. A skewed T-helper 1 (Th1) SARS-CoV-2–specific T-cell response and higher percentages of IgG-expressing memory B cells were observed postvaccination compared to postinfection [45]. We assessed the functional and phenotypic features of SARS-CoV-2–specific CD4^+^/CD8^+^ T cells prevaccination in cases, comparing these in individuals with or without BIs. We show trends toward increased SARS-CoV-2–specific CD8^+^ T cells targeting S2, N, M, and NSP3B in individuals without BI, with differences significantly higher only for M, suggesting a protective role for these cells. Similarly, M-specific CD4^+^ T cells are present at higher magnitudes in individuals without BI. Additionally, this group displayed mainly IFN-γ and CD107a-producing M-specific CD4^+^ T cells suggesting a protective role for cytotoxic Th1 cells in COVID-19. This highlights how a granular analysis of CD4^+^/CD8^+^ T-cell responses may inform T-cell features associated with protection, which is critical for an integrated (incorporating T cells and antibodies) definition of correlates/determinants of protection to SARS-CoV-2 and potentially other viral infections.

In addition to qualitatively changing responses, SARS-CoV-2 infection primes the immune system with a broader range of antigens than vaccination. Anti-N seropositivity strongly associated with protection against reported BIs in our cohort, corroborating previous estimates that it affords participants with approximately 80% protection against SARS-CoV-2 reinfection for the subsequent 8 months [46]. In those experiencing BIs, anti-N seropositivity was also associated with shorter durations and reduced viral load [35]. Evidence suggests that COVID-19 vaccination drives differential responses to subsequent SARS-CoV-2 infection, boosting those primed by the vaccine over new responses to the broader repertoire of proteins in the virus [35, 47, 48]. Seroconversion to N upon SARS-CoV-2 infection occurred at a higher rate in placebo-vaccinated participants (93%) compared to mRNA-1273 recipients (40%) [47]. Additionally, SARS-CoV-2 BIs elicit anti-N IgG at lower rates in infection-naive individuals compared to those with a history of prevaccination COVID-19 [35, 48], suggesting that the order in which hybrid immunity is achieved may be important.

LFT testing of the wider ALSPAC cohort highlighted an age-related difference in SARS-CoV-2–specific anti-N positivity rates. By spring 2022, all UK adults had been offered 3 vaccine doses, and this was reflected in comparable anti-S positivity rates of 93.0% and 96.0% in the G0 and G1 cohorts, respectively. However, only 50.3% of the older generation was anti-N positive compared with 67.1% of younger adults. These values may underestimate SARS-CoV-2 infection rates in older adults due to the lower N-seroconversion rates in postvaccination infections. However, they accurately reflect a lack of potentially protective anti-N antibodies. Prioritizing older adults for early vaccination and encouraging shielding behaviors may have consequences for the breadth and durability of immunity obtained by reducing prevaccination infection rates and overall N-seroconversion. Rapidly vaccinating older adults reduced severe disease and mortality, and on a population level these benefits are unlikely to be outweighed by the benefits of broader infection-primed responses. Instead, this highlights the importance of designing novel vaccines that prime responses to additional antigens to generate more robust and sustained protection.

Our BI susceptibility analysis should be interpreted in the context of the study design limitations. First, participants were predominantly White, with a low incidence of immunosuppressive disease. Second, analysis was limited to those with post–second vaccination data and may not be fully representative. Third, BI rates in cases/controls may be influenced by differences in exposure risk or test-seeking behaviors, which could not be accounted for. Fourth, N-protein serology does not offer 100% sensitivity for detecting new SARS-CoV-2 cases; therefore, undetected infections could have resulted in misclassifying participants as controls. Fifth, ICS was only performed on samples with positive ELISpot results and may therefore not be representative of the whole study population. Sixth, ex vivo ELISpot results may not accurately reflect the proliferative potential of T cells. Seventh, samples were not collected at the point of BI and therefore could not be confirmed virologically, nor the SARS-CoV-2 sequences/variant-lineages determined, preventing subanalysis by variant type. The BI-monitoring period covered the period of Delta/Omicron being the predominant circulating variants, and variant-specific thresholds for protection may differ and warrant further investigation. Finally, rather than being directly protective, reported markers may act as proxies for undetermined variables that were not measured. These may include nasal mucosal antibody and T-cell responses, which were identified as important for protection in human challenge studies [49].

In summary, our data enrich the evidence of broader immune responses to infection and vaccination beyond the comparatively well-characterized anti-S IgG and nAb levels as markers of protection against SARS-CoV-2 BIs. Additionally, our findings support the notion that it is not only the magnitude of T-cell responses generated by hybrid immunity that improves protection, but also their breadth and quality. This reinforces the need for next-generation vaccines that elicit hybrid-mimicking immune responses for more robust and sustained protection. Better understanding the immune markers correlated with protection will also be important for rapidly validating new vaccines at a point in the pandemic when placebo-controlled vaccine trials are no longer practical.

## Supplementary Material

jiaf246_Supplementary_Data

## Supplementary Data

Supplementary materials are available at *The Journal of Infectious Diseases* online (http://jid.oxfordjournals.org/). Supplementary materials consist of data provided by the author that are published to benefit the reader. The posted materials are not copyedited. The contents of all supplementary data are the sole responsibility of the authors. Questions or messages regarding errors should be addressed to the author.

## Notes

***Author contributions.*** Conceptualization: A. F., N. J. T., L. W., A. H., L. R., and A. T. Methodology: H. E. B., M. S., O. F., E. M., E. O., R. E. M., A. T., and A. H. Validation: H. E. B., M. S., O. F., E. M., E. O., A. T., and A. F. Formal analysis: H. E. B., M. S., O. F., R. E. M., M. K., and A. H. Investigation: H. E. B., M. S., O. F., E. M., K. E., E. O., B. H., D. D., A. T., and B. M.-A. Resources: K. G., N. D. B., P. K., A. B., I. B., and A. M. T. Data curation: K. N., F. R., H. E. B., and M. S. Writing–original draft: H. E. B., M. S., L. W., A. H., and L. R. Writing–review and editing: O. F., E. O., R. E. M., P. K., A. G., A. F., N. J. T., and A. H. Visualization: H. E. B., M. S., and O. F. Supervision: A. M. T., I. B., A. D., A. G., A. F., L. W., A. H., and L. R. Project administration: J. O., S. R., L. M., L. W., A. H., and L. R. Funding acquisition: A. F., S. R., L. M., N. J. T., L. W., A. H., and L. R.

***Acknowledgments.*** We are extremely grateful to all of the families who took part in this study; the midwives for their help in recruiting them; and the entire ALSPAC team, which includes interviewers, computer and laboratory technicians, clerical workers, research scientists, volunteers, managers, receptionists, and nurses. We also acknowledge the assistance of Dr Andrew Herman, Helen Rice, and the University of Bristol Faculty of Health and Life Sciences Flow Cytometry Facility. This work was undertaken with support from the Elizabeth Blackwell Institute (EBI) Mechanisms to Populations Research Strand that promotes interdisciplinary research between fundamental bioscience and population health. The support from the EBI and the University's Alumni and Friends funded the purchase of the CTL ELISpot reader and ELISA microplate washer and the ELISpot peptide pools used in this study. We thank the Bristol UNCOVER group for helpful discussions on assay development and the interpretation of results.

***Disclaimer.*** The funders had no role in the study design, data collection, data analysis, preparation of the manuscript, or decision to publish.

***Financial support.*** This work was funded by UK Research and Innovation (UKRI) through the UKRI rapid response call for COVID (MR/V028448/1). This work was supported by the Elizabeth Blackwell Institute, University of Bristol, with funding from the Wellcome Trust ISSF3 (204813/Z/16/Z), and the University's Alumni and Friends (to L. W., L. R., A. H., A. D., A. F., and O. F.). N. J. T. is the principal investigator of the Avon Longitudinal Study of Parents and Children (ALSPAC) (Medical Research Council [MRC] and WT 217065/Z/19/Z); is supported by the University of Bristol National Institute for Health and Care Research Biomedical Research Centre (BRC-1215-2001) and the Medical Research Council (MRC) Integrative Epidemiology Unit (MC_UU_00011/1); and works within the Cancer Research UK Integrative Cancer Epidemiology Programme (C18281/A29019). M. K. is supported by the MRC (MR/W021315/1). M. S. is supported by the Academy of Medical Sciences (Springboard Award SB007/100173). The MRC and the Wellcome Trust (grant ref: 217065/Z/19/Z) and the University of Bristol provide core support for ALSPAC. This publication is the work of the authors; L. R., A. H., L. W., H. E. B., and M. S. serve as guarantors for the contents of this article. A comprehensive list of grant funding is available on the ALSPAC website (http://www.bristol.ac.uk/alspac/external/documents/grant-acknowledgements.pdf). This research was specifically funded by the Wellcome Trust and the MRC (grant 102215/Z/13/Z).
