# Correction to: Hybrid B- and T-Cell Immunity Associates With Protection Against Breakthrough Infection After Severe Acute Respiratory Syndrome Coronavirus 2 Vaccination in Avon Longitudinal Study of Parents and Children (ALSPAC) Participants

**DOI:** 10.1093/infdis/jiag161

**Published:** 2026-03-18

This is a correction to: Holly E Baum, Marianna Santopaolo, Ore Francis, Emily J Milodowski, Katrina Entwistle, Elizabeth Oliver, Benjamin Hitchings, Divya Diamond, Amy C Thomas, Ruth E Mitchell, Milla Kibble, Kapil Gupta, Natalie Di Bartolo, Paul Klenerman, Anthony Brown, Begonia Morales-Aza, Jennifer Oliver, Imre Berger, Ash M Toye, Adam Finn, Anu Goenka, Andrew D Davidson, Susan Ring, Lynn Molloy, Melanie Lewcock, Kate Northstone, Firona Roth, Nicholas J Timpson, Linda Wooldridge, Alice Halliday, Laura Rivino, Hybrid B- and T-Cell Immunity Associates With Protection Against Breakthrough Infection After Severe Acute Respiratory Syndrome Coronavirus 2 Vaccination in Avon Longitudinal Study of Parents and Children (ALSPAC) Participants, *The Journal of Infectious Diseases*, Volume 232, Issue 2, 15 August 2025, Pages e327–e340, https://doi.org/10.1093/infdis/jiaf246

A grant number in the final sentence of the section ***Financial support***. Has been corrected to read: “This research was specifically funded by the Wellcome Trust and the MRC (grant 102215/Z/13/Z).” instead of: “This research was specifically funded by the Wellcome Trust and the MRC (grant 102215/2/13/2).”

