# Supplementary material for: Hybrid B- and T-Cell Immunity Associates With Protection Against Breakthrough Infection After Severe Acute Respiratory Syndrome Coronavirus 2 Vaccination in Avon Longitudinal Study of Parents and Children (ALSPAC) Participants
Source: J Infect Dis. 2025 May 20;232(2):e327–40. doi: 10.1093/infdis/jiaf246 (PMC12349941; doi:10.1093/infdis/jiaf246)
Supplement: jiaf246_Supplementary_Data [file jiaf246_supplementary_data.zip › Baum_ALSPAC_JID_SupplementaryText.docx]

**EXTENDED METHODS**

***ALSPAC***

The Avon Longitudinal Study of Parents and Children (ALSPAC) is a birth cohort study (1–4). Pregnant women resident in Avon, UK with expected dates of delivery between 1st April 1991 and 31st December 1992 were invited to take part. A total of 14,541 pregnancies were initially enrolled, with 13,988 children who were alive at 1 year of age. ALSPAC now comprises three generations: the original pregnant women with the biological fathers and other carers/partners (G0), the cohort of index children (G1), and the offspring of the index children (G2). This has generated a wealth of biological, genetic and phenotypic data spanning the lifetime of these individuals. Including additional participants recruited in the interim period, the total sample size for analyses using any data collected after the age of seven is 15,447 pregnancies, resulting in 15,658 foetuses. Of these 14,901 children were alive at 1 year of age. 12,113 G0 partners have been in contact with the study, of which 3,807 are currently enrolled. Please note that the study website contains details of all the data that are available through a fully searchable data dictionary and variable search tool (http://www.bristol.ac.uk/alspac/researchers/our-data/). From the beginning of the COVID-19 pandemic, ALSPAC sought to utilise their unique expertise and infrastructure to contribute to SARS-CoV-2 research efforts through collection of biological samples and questionnaire data from their cohort of well characterised participants.

***Study design***

In October 2020, ALSPAC undertook 5200 serological SARS-CoV-2 spike-specific lateral flow tests (Fortress Diagnostics, Antrim Northern Ireland) on G0 and G1 cohort participants (5). Participants with evidence of a previous SARS-CoV-2 infection based on a positive IgG result on this LFT, and/or a positive SARS-CoV-2 PCR result from linked UK Health Security Agency (UKHSA) data, were invited to take part in the study (n=124). Two control groups with negative serological results, and no documented positive PCR test, were recruited alongside these participants. The first control group (n=93) were also age, sex, and symptom (anosmia) matched to those with a history of SARS-CoV-2 infection. Participants in the second control group (n=103) were selected on the basis of not having reported anosmia. Full details of the recruitment methodology, alongside a detailed characterisation of the cohort at clinic-1 (pre-vaccination), are described in Mitchell *et al*. In brief, participants attended up to 3 clinics in December 2020 (clinic-1), March 2021 (clinic-2) and June 2021 (clinic-3) where they provided venous blood and saliva samples. Additional participants were recruited at clinics 2 and 3 to maintain numbers and account for those who withdrew from the study. Health and lifestyle information was gathered through online questionnaires. For the purposes of the analyses presented in this study, the two control groups have been combined as no significant differences in baseline antibody or T-cell measures were detected between the two original groups (6).

***Ethics***

Ethical approval for the study was obtained from the ALSPAC Ethics and Law Committee and the Local Research Ethics Committees (NHS REC 20/HRA/4854). Consent for biological samples was collected in accordance with the Human Tissue Act (2004). Informed consent for the use of data collected via questionnaires and clinics was obtained from participants following the recommendations of the ALSPAC Ethics and Law Committee at the time.

At age 18, study children were sent 'fair processing' materials describing ALSPAC’s intended use of their health and administrative records and were given clear means to consent or object via a written form. Data were not extracted for participants who objected, or who were not sent fair processing materials. Ethical approval was obtained from the ALSPAC Law and Ethics committee and local research ethics committees (NHS Haydock REC 10/H1010/70).

***Sample collection and processing***

Peripheral blood mononuclear cells (PBMCs) were obtained from up to 3 x 10 ml EDTA tubes per participant using standard density gradient separation techniques. Briefly, samples were kept at room temperature for up to 3 hours after drawing blood. Blood was diluted 1:1 with Phosphate Buffered Saline (PBS) containing 1% Fetal calf serum (FCS). Diluted blood was separated using a Ficoll gradient, centrifuging at 1000g for 10 mins at room temperature. PBMCs were washed in PBS/1% FCS, centrifuging at 700g for 10 mins at room temperature, and washed again with PBS/1% FCS, centrifuging at 400g for 10 mins at room temperature. Cell pellets were resuspended in freezing mix (90% FCS/10% DMSO) at a concentration of 13-15x10^6^ cells per mL. Cells were frozen overnight in an alcohol bath to control freezing rate. PBMCs were then transferred to liquid nitrogen cryotanks for long-term storage. Serum tubes were left to clot, centrifuged at 1,500g for 10 mins at 18-25°C, then serum was removed, aliquoted, and stored at -80 °C.

Participants provided neat saliva directly into a sterile collection tube. Particulate matter was removed by centrifugation at 13,000g for 10 minutes. Samples were heat-inactivated at 56°C for 30 minutes prior to ELISA analysis.

***ELISAs***

SARS-CoV-2-specific anti-Spike (S) IgA and IgG in serum and saliva were measured by ELISA. Samples were run in duplicate at a single optimised dilution and reported in BAU/mL following calibration of an internal standard to the WHO/NIBSC reference control. Serum anti-nucleocapsid (N) and anti-S pan Ig were utilised as screening ELISAs to report a positive/negative result for previous SARS-CoV-2 infection with data presented as normalised optical density (OD) measurement relative to an internal control. Thresholds for positivity were calculated on a large sample of PCR-confirmed and pre-pandemic samples. Full details of these methods are published for serum (7), and saliva (8), respectively.

***Synthetic peptides***

Peptides used for PBMC stimulations in the ELISpot assays are listed in Supplementary Table 4. 15-mer peptides overlapping by 10 amino acids and spanning the sequences of the SARS-CoV-2 S, N, Membrane (M) and Envelope (E) protein were purchased from Mimotopes (Australia). The purity of the peptides was >80%. 15-18mer peptides overlapping by 10 amino acids and spanning sequences of SARS-CoV-2 NSP1-16, ORF3, ORF6, ORF7 and ORF8 were also purchased). The purity of the peptides was ~70%. The combination of peptides and identity of the peptide pools are described in Supplementary Table 4. For PBMC stimulation in ELISpot assays, pools of a maximum of 127 peptides were added per well as follows: Spike was divided into 2 pools (S1; 126 peptides and S2; 127 peptides); for smaller peptide regions, peptides were combined in the same peptide pools as follows: NSP1 and NSP2 (NSP1+2), NSP5 and NSP6 (NSP5+6), NSP15 and NSP16 (NSP15+16). All other peptides were pooled into one mixture and tested in individual ELISpot wells.

***Enzyme-Linked Immunosorbent Spot assay (ELISpot)***

Human IFN-γ ELISpot assays were performed using a Human IFN-γ ELISpot BASIC kit (Mabtech). MSIP4W10 PVDF plates (Millipore) were coated with capture antibody (mAb-1-D1K; 15 μg/mL) and incubated overnight at 4 °C in carbonate bicarbonate buffer (Sigma Aldrich). Cryopreserved PBMC were thawed then rested at 37 °C/5% CO_2_ for 5-6 hours. Coated plates were washed 5 times in sterile PBS and blocked for 1-2 hours using R10 medium (0.2 µm filtered RPMI 1640 medium supplemented with 10 % FBS, 2 mM glutamine, penicillin ((100 units/ml) and streptomycin (100 μg/ml)). 4 x 10^5^ PBMCs were added to each well in the plate, with or without peptide pools (as indicated) in a total assay volume of 100 µl in R10. PBMC incubated with R10 medium alone were used as negative (unstimulated) controls and were performed in duplicate. Peptide pools spanning S1, S2, M, N, E, ORF1 (NSP1+2, NSP3A, NSP3B, NSP3C, NSP4, NSP5+6, NSP7-11, NSP12A, NSP12B, NSP13, NSP14, NSP15+16), ORF3, ORF6, ORF7 and ORF8 were used at a final concentration of 2 µg/ml. PBMCs from cases were tested against all the above peptide pools, while PBMCs from controls were tested against the following peptide pools only: S1, S2, M, N, NSP3B, NSP12A, NSP12B, NSP7-11, NSP13 and NSP15+16. PBMC stimulated with anti-CD3 antibody (Mabtech, Mab CD3-2; final concentration 0.1% v/v) were used as a positive control for each participant, with 1-4 x 10^5^ PBMCs used per well. Positive control and peptide stimulated wells were performed in singlet. Plates were incubated for 16-18 hours at 37°C/5% CO_2_ then developed as per manufacturer's instructions. Developed plates were protected from light and air-dried for 48 hours before image acquisition using a CTL ImmunoSpot S6 Ultra-V Analyzer. Spot forming units (SFU) were calculated using the ImmunoSpot S6 Ultra-V Analyzer Basic Count function after image acquisition using optimised counting parameters that were applied across all participants. Spot counts were enumerated for each peptide pool by subtraction of average background (calculated from duplicate unstimulated wells). Counts were expressed as SFU per million (10^6^) PBMC after multiplication by 2.5 following background subtraction. Negative values after background subtraction were adjusted to zero (9). Participants were excluded if the spot count in unstimulated wells exceeded 95 SFU per million PBMC or if no spots were observed in the positive control wells. Where spot formation was too dense to accurately enumerate using standardised counting parameters (TNTC; too numerous to count), affected wells were excluded unless contemporaneous assessment of IFN-γ production by flow cytometry confirmed antigen specific response. In these cases, TNTC values were given the raw value equivalent to the largest spot count accurately counted for a peptide pool (320 SFU per well).

***Intracellular Cytokine Staining (ICS)***

PBMCs were thawed and rested overnight in AIMV 2% FCS then incubated with or without peptide pools from SARS-CoV-2 S1/S2, M, N, NSP3B (all 1 μg/ml), or with PMA/ionomycin (PMA 10 ng/ml, ionomycin 100 ng/ml, Sigma-Aldrich) for 5 hr at 37°C in the presence of brefeldin A (BD, 5 μg/ml). To assess degranulation, anti CD107a-FITC antibody was added to the cells at the beginning of the stimulation. Cells were stained with a viability dye Zombie Aqua (BioLegend) for 10 min at room temperature and then with antibodies targeting surface markers (20 min 4°C, diluted in PBS 1% BSA; Sigma-Aldrich). Cells were fixed overnight in eBioscience Foxp3/Transcription factor fixation/permeabilization buffer (Invitrogen), and intracellular staining was performed for detection intracellular cytokines, including IFN-γ, TNF-α, IL-2 and MIP1β (30 min 4°C). Four samples were excluded from further CD4^+^ T-cell analysis due technical issues. Data were acquired on a BD LSR Fortessa X20 and analysed using FlowJo software v10.8.1. Results were obtained after subtraction of the values in the corresponding unstimulated well. ICS was performed on samples from 69 individuals in the case group, selected based on having a detectable T-cell response by IFN-γ ELISpot at baseline (i.e. clinic one) for at least one of the SARS-CoV-2 peptide pools tested and for whom we had additional cryopreserved PBMC vials available at baseline.

***Pseudoneutralisation***

Serum neutralisation was expected to positively correlate with levels of anti-Spike antibody binding results. Accordingly, all samples from participants in the case group corresponding to anti-Spike pan-Ig ELISA results above a normalised threshold of 0.5 were included. For pseudovirus assays, Wuhan-Spike-harbouring pseudovirus (luciferase-expressing vesicular stomatitis virus, VSV-S-FLuc) was generated and used to assess serum antibody neutralisation of VSV-S-FLuc entry into Vero ACE2 TMPRSS2 (VAT) cells as described previously (7) serum dilutions starting at 1/40 followed by eight 2.5-fold titrations were plated in triplicate in 96-well plates, alongside three wells each of 1/25 dilutions of known neutralising and non-neutralising controls (corresponding to 16,000 RLU of luminescence when mixed with VSV-S-FLuc). WT Wuhan spike pseudotyped VSV was added to each well and incubated for an hour. Well-mixtures were added to black, microscopy 96-well plates, seeded with 10,000 Vero ACE2 TMPRSS2 (VAT) cells per well. Luminescence measurements were taken 16 hours after infection, using the ONE-Glo Luciferase Assay System.

***Data analysis***

Study data were collected and managed using REDCap (Research Electronic Data Capture) electronic data capture tools hosted at the University of Bristol. REDCap is a secure, web-based software platform designed to support data capture for research studies.

Participant data was excluded from the analysis if it was subsequently established that a participant was vaccinated as part of an unlicensed COVID-19 vaccine trial prior to enrolment in this study. In order to focus on the immune responses to COVID-19 vaccines, data was also removed if no corresponding information provided on the COVID-19 vaccination status of the participant at a particular clinic visit. Where a participant provided samples at more than one clinic after a specified number of vaccine doses (including pre-vaccination), only the data from the earliest sampling clinic was included in the analysis.

Statistical analyses were performed using R Studio (v4.3.0), and GraphPad Prism (version 10.01). Unpaired comparisons across multiple groups were done with the Kruskal-Wallis test with Dunn's post-test for multiple comparisons. Pairwise correlations were assessed with Spearman's rank-order correlation (r_s_). Correlation coefficients were interpreted as: weak (r_s_=0.20-0.39), moderate (r_s_=0.40-0·59), strong (r_s_=0.60-0.79), or very strong (r_s_=0.80-1.00). The following adjusted P value thresholds were used for data visualisation: P≤0.05 (*), P≤0.01 (**), P≤0.001 (***), P≤0.0001 (****). To facilitate the presentation of data which included zero counts on log scales, zero counts were plotted as 1 (or the minimum y-axis baseline, if lower) for visualisation purposes only – all statistical analyses were performed on the raw data values). Scaled immunological data were used to generate the heatmap using the *pheatmap* package within R, which uses the Euclidean distance as the similarity measure and clusters samples based on the 'complete' method. To aid visualisation of clusters, the heatmap was split into the three main clusters of both participants and assays.

1. Northstone K, Ben Shlomo Y, Teyhan A, Hill A, Groom A, Mumme M, et al. The Avon Longitudinal Study of Parents and children ALSPAC G0 Partners: A cohort profile. Wellcome Open Res. 2023 Jan 24;8:37.

2. Fraser A, Macdonald-wallis C, Tilling K, Boyd A, Golding J, Davey smith G, et al. Cohort profile: The avon longitudinal study of parents and children: ALSPAC mothers cohort. Int J Epidemiol. 2013 Feb;42(1):97–110.

3. Boyd A, Golding J, Macleod J, Lawlor DA, Fraser A, Henderson J, et al. Cohort profile: The ’Children of the 90s’-The index offspring of the avon longitudinal study of parents and children. Int J Epidemiol. 2013 Feb;42(1):111–27.

4. Northstone K, Lewcock M, Groom A, Boyd A, Macleod J, Timpson N, et al. The Avon Longitudinal Study of Parents and Children (ALSPAC): an update on the enrolled sample of index children in 2019. Wellcome Open Res. 2019;4.

5. Northstone K, Smith D, Bowring C, Hill A, Hobbs R, Wells N, et al. The Avon Longitudinal Study of Parents and Children - A resource for COVID-19 research: Home-based antibody testing results, October 2020. Wellcome Open Res. 2021;6.

6. Mitchell RE, Kibble M, Santopaolo M, Milodowski E, Baum HE, Francis O, et al. SARS-CoV-2 memory response in non-hospitalised cases: immunology in the context of a population-based cohort study. Wellcome Open Res [Internet]. 2024 Oct 11;9:580. Available from: https://wellcomeopenresearch.org/articles/9-580/v1

7. Halliday A, Long AE, Baum HE, Thomas AC, Shelley KL, Oliver E, et al. Development and evaluation of low-volume tests to detect and characterize antibodies to SARS-CoV-2. Front Immunol. 2022 Nov 9;13.

8. Thomas AC, Oliver E, Baum HE, Gupta K, Shelley KL, Long AE, et al. Evaluation and deployment of isotype-specific salivary antibody assays for detecting previous SARS-CoV-2 infection in children and adults. Communications Medicine [Internet]. 2023 Mar 15;3(1):37. Available from: https://www.nature.com/articles/s43856-023-00264-2

9. Swadling L, Diniz MO, Schmidt NM, Amin OE, Chandran A, Shaw E, et al. Pre-existing polymerase-specific T cells expand in abortive seronegative SARS-CoV-2. Nature. 2022 Jan 6;601(7891):110–7.
