# Supplementary figures and images for: Hybrid B- and T-Cell Immunity Associates With Protection Against Breakthrough Infection After Severe Acute Respiratory Syndrome Coronavirus 2 Vaccination in Avon Longitudinal Study of Parents and Children (ALSPAC) Participants
Source: J Infect Dis. 2025 May 20;232(2):e327–40. doi: 10.1093/infdis/jiaf246 (PMC12349941; doi:10.1093/infdis/jiaf246)

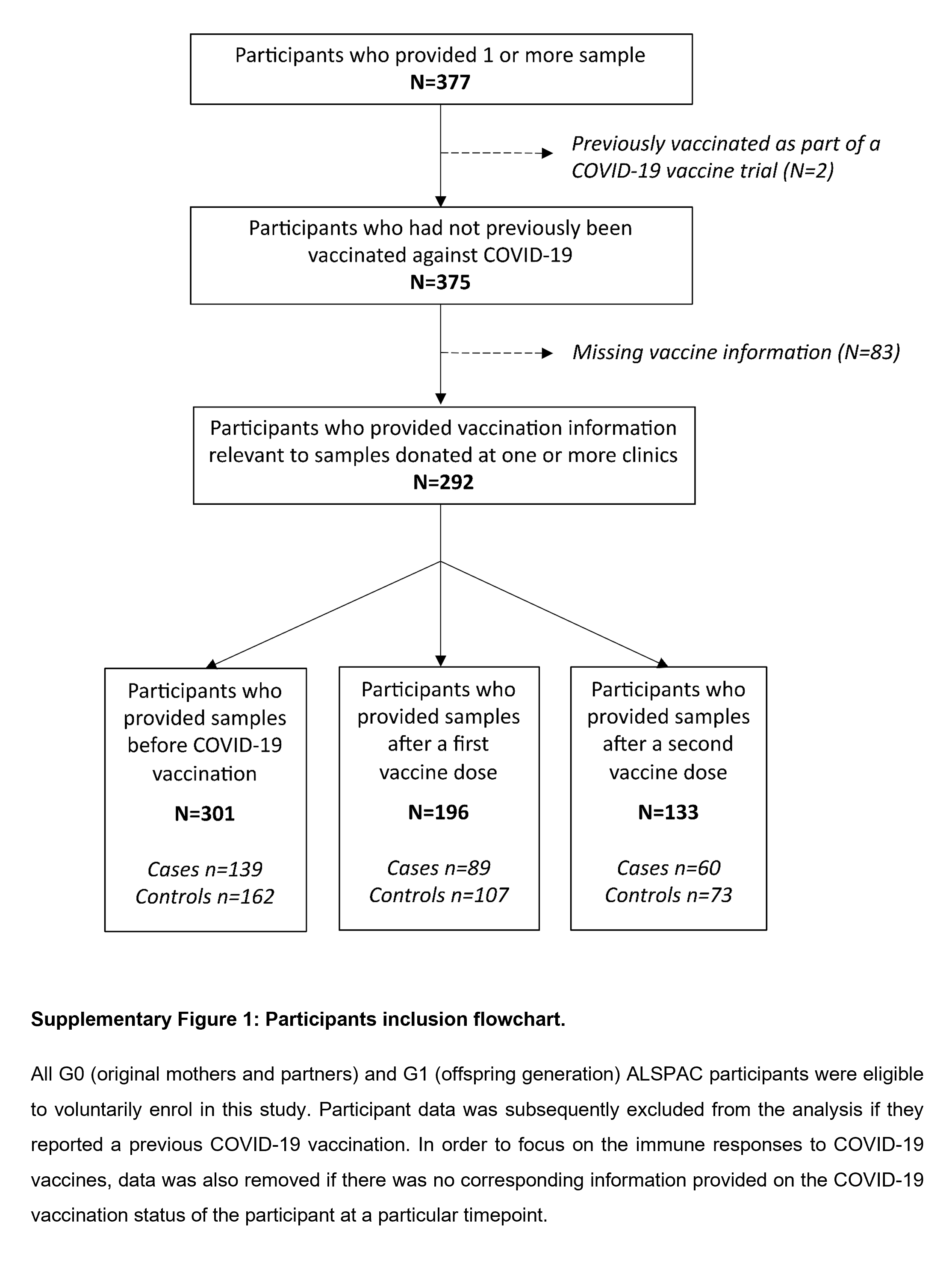

Supplement: jiaf246_Supplementary_Data [file jiaf246_supplementary_data.zip › Supplementary_Figure_1.png]

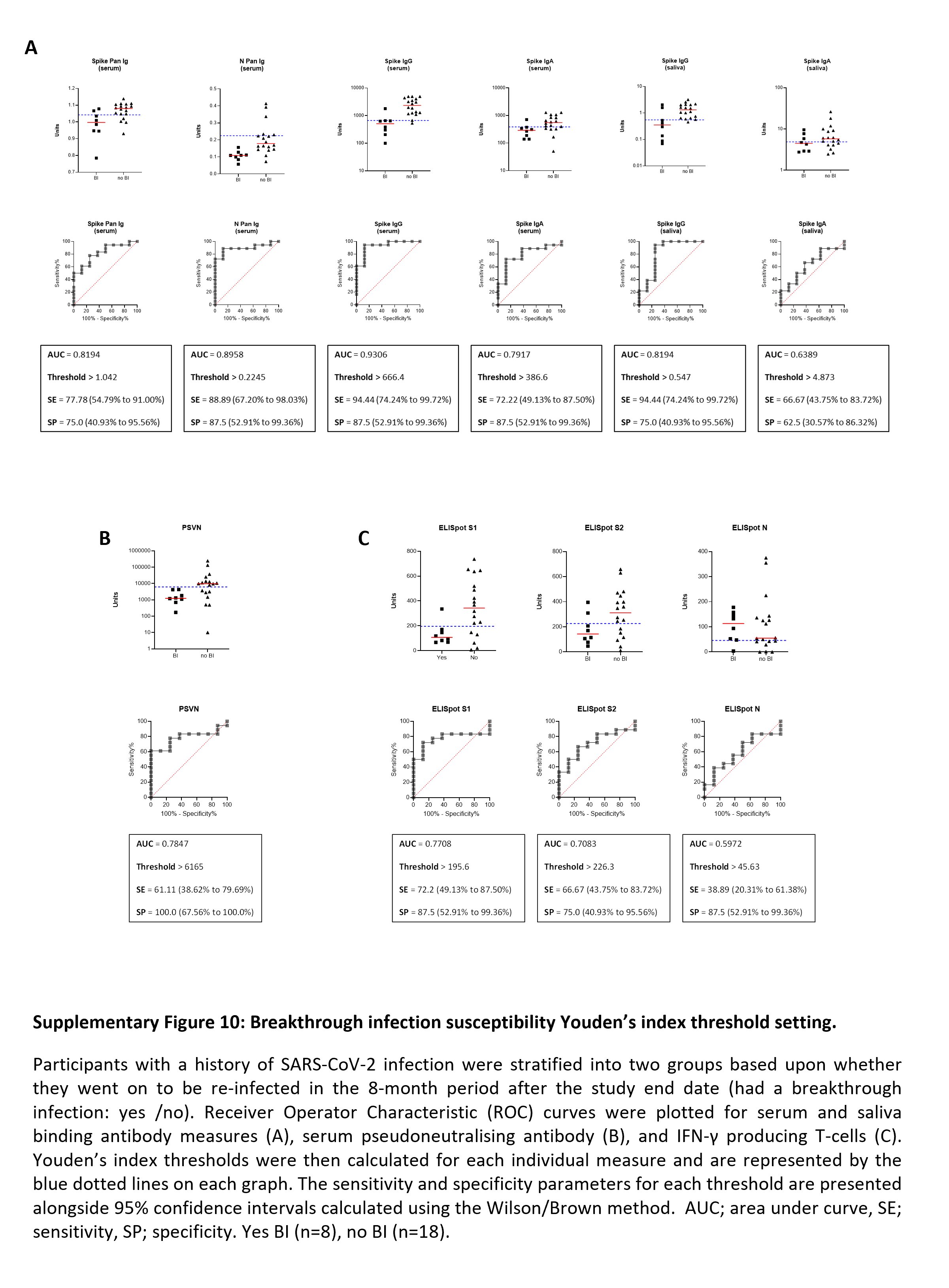

Supplement: jiaf246_Supplementary_Data [file jiaf246_supplementary_data.zip › Supplementary_Figure_10.png]

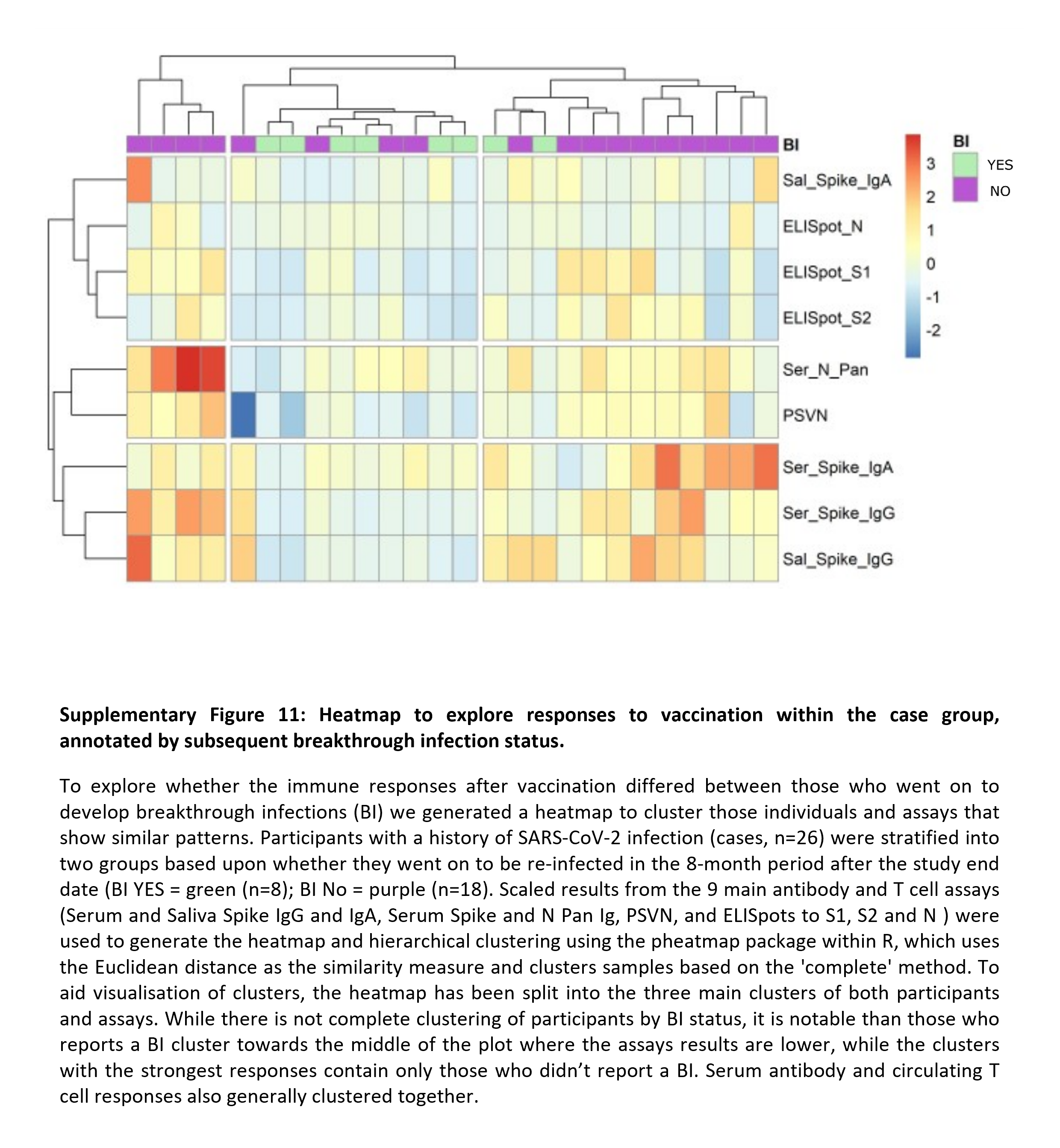

Supplement: jiaf246_Supplementary_Data [file jiaf246_supplementary_data.zip › Supplementary_Figure_11.png]

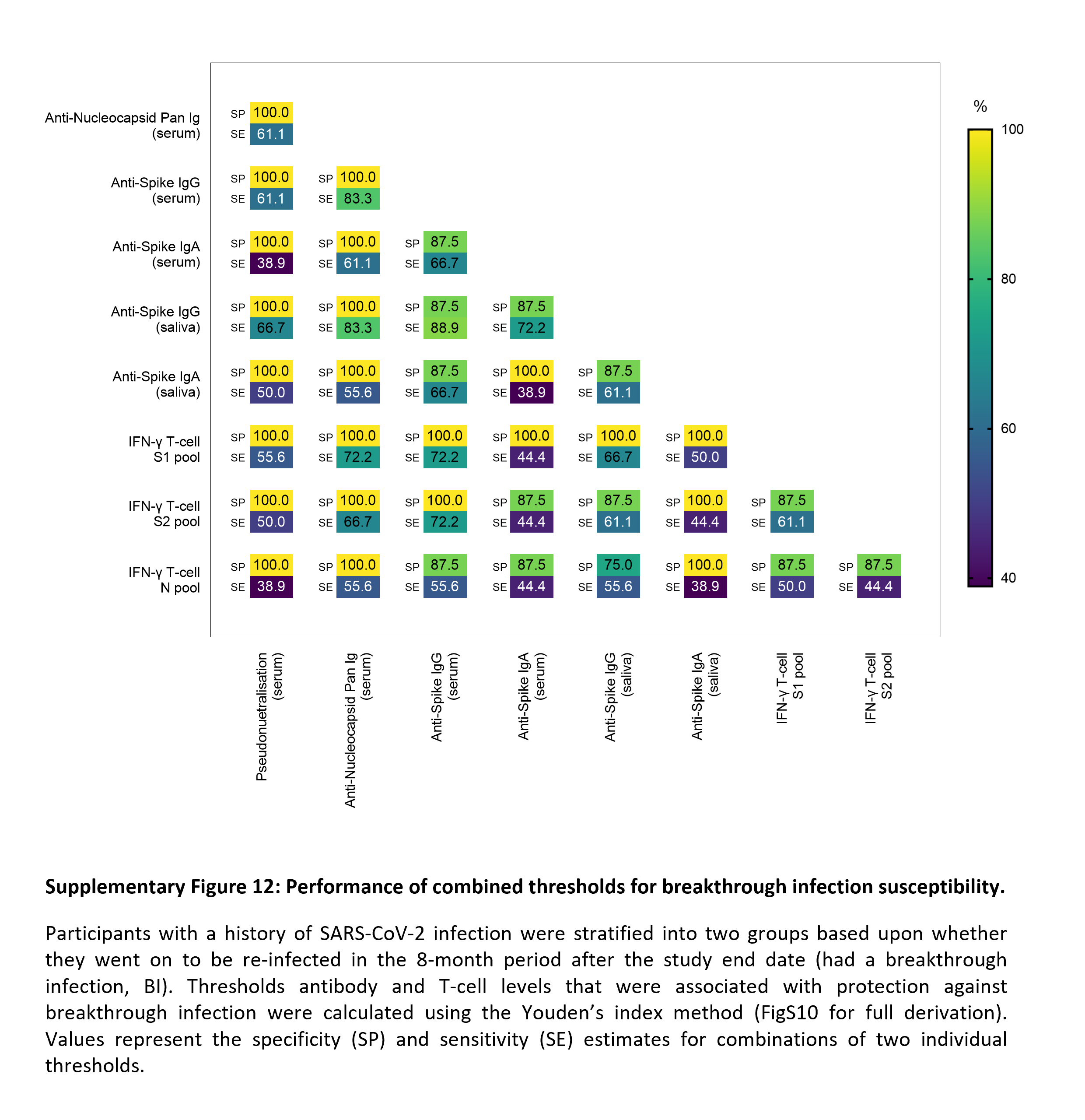

Supplement: jiaf246_Supplementary_Data [file jiaf246_supplementary_data.zip › Supplementary_Figure_12.png]

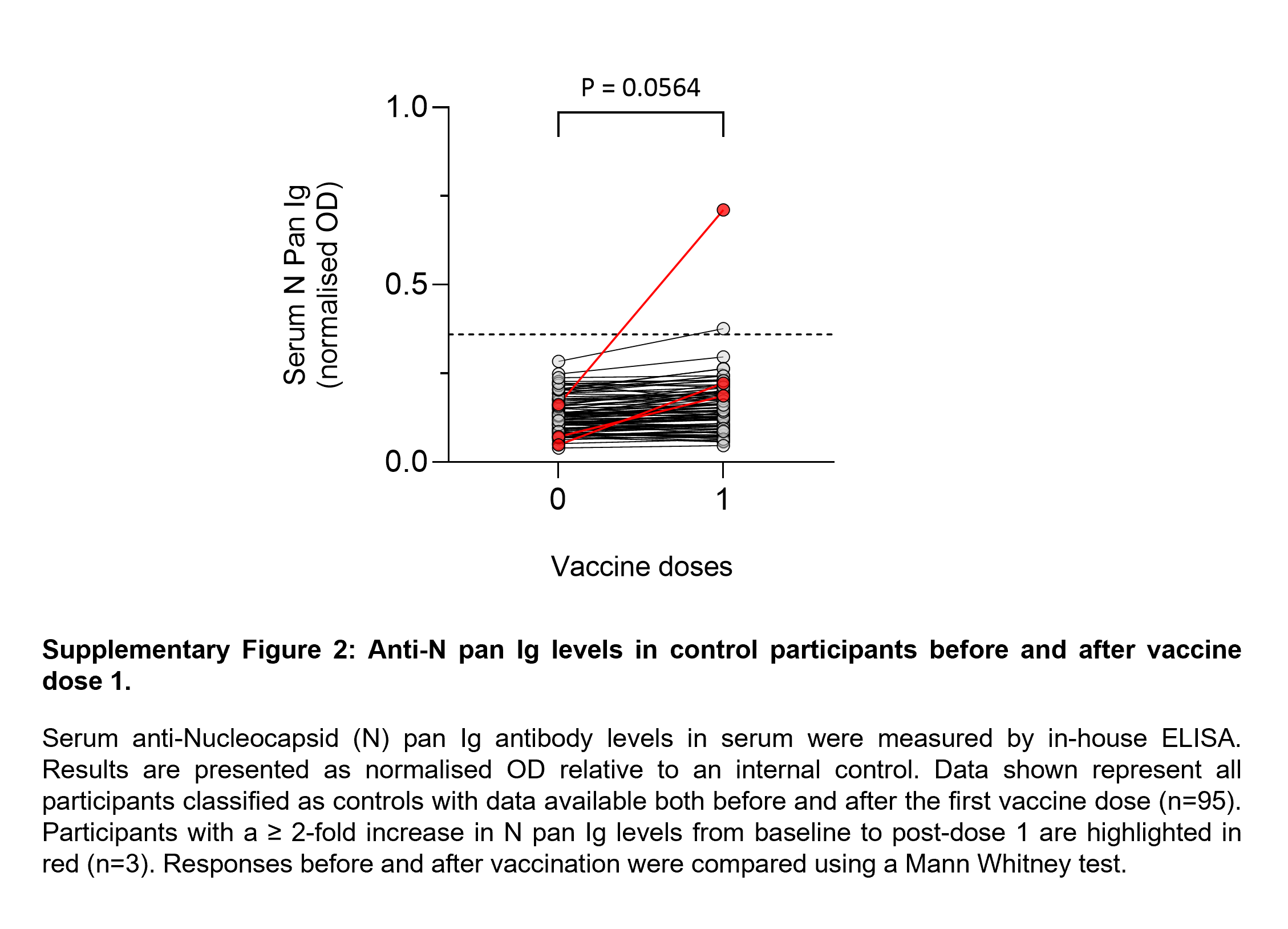

Supplement: jiaf246_Supplementary_Data [file jiaf246_supplementary_data.zip › Supplementary_Figure_2.png]

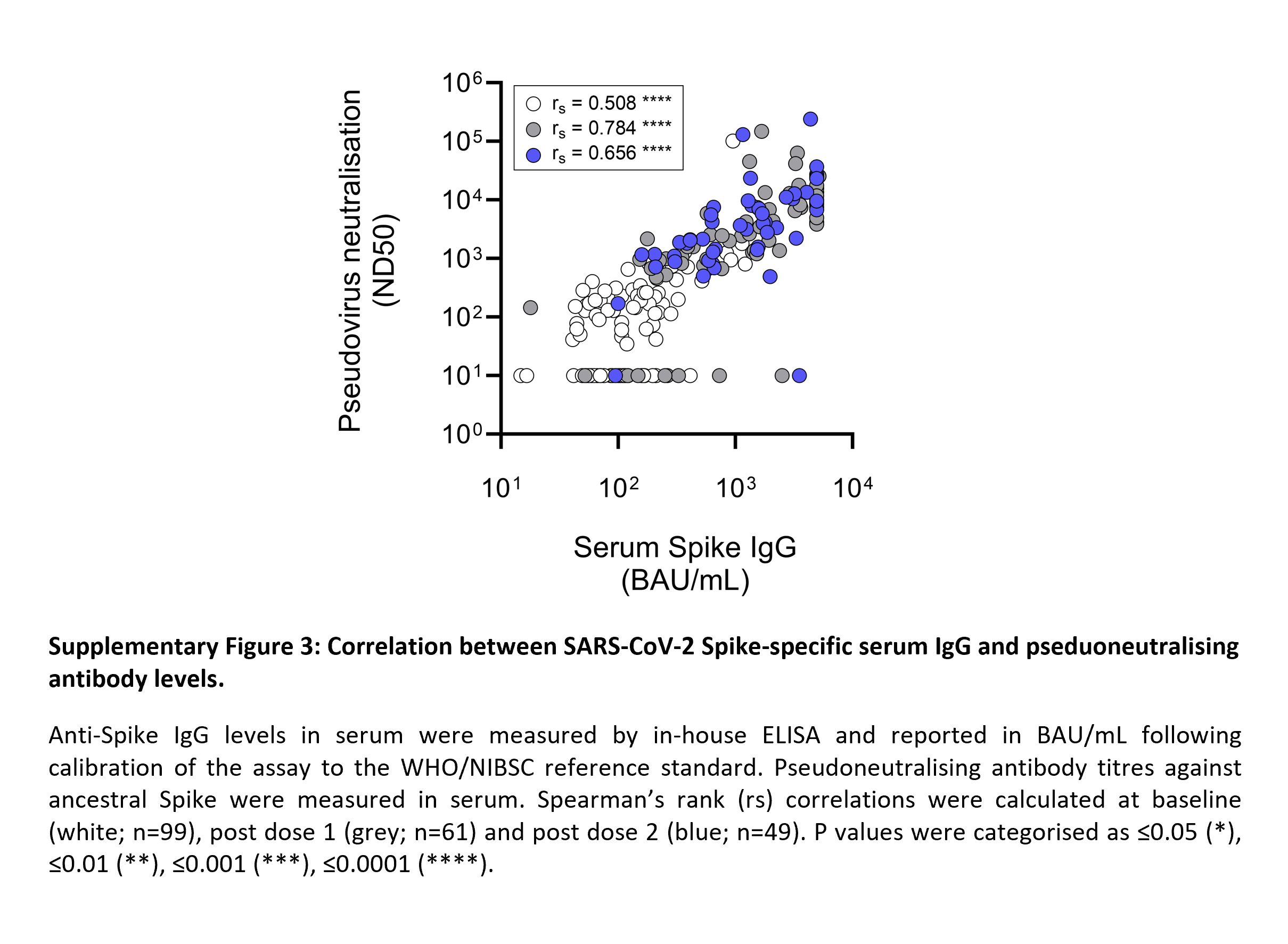

Supplement: jiaf246_Supplementary_Data [file jiaf246_supplementary_data.zip › Supplementary_Figure_3.png]

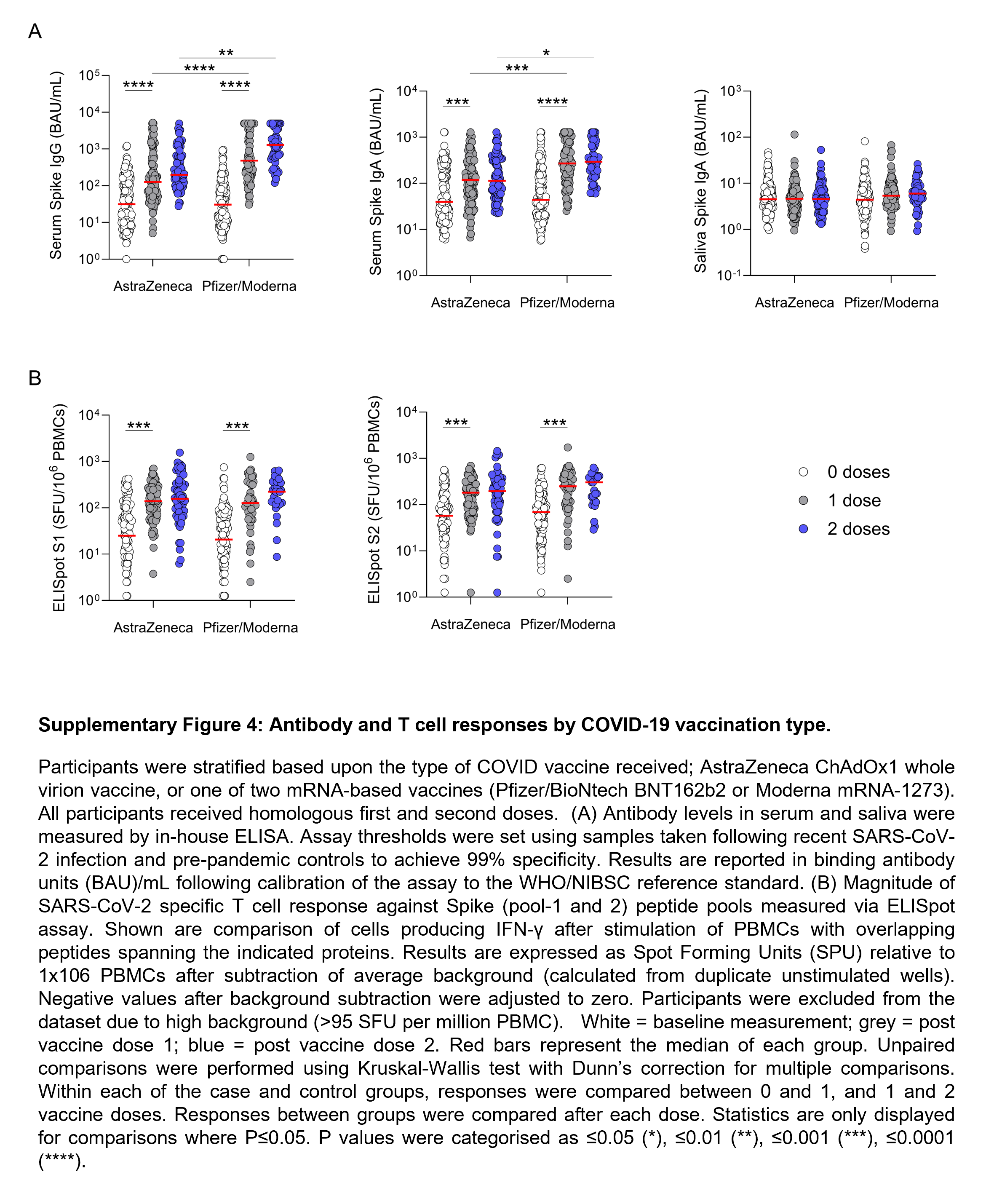

Supplement: jiaf246_Supplementary_Data [file jiaf246_supplementary_data.zip › Supplementary_Figure_4.png]

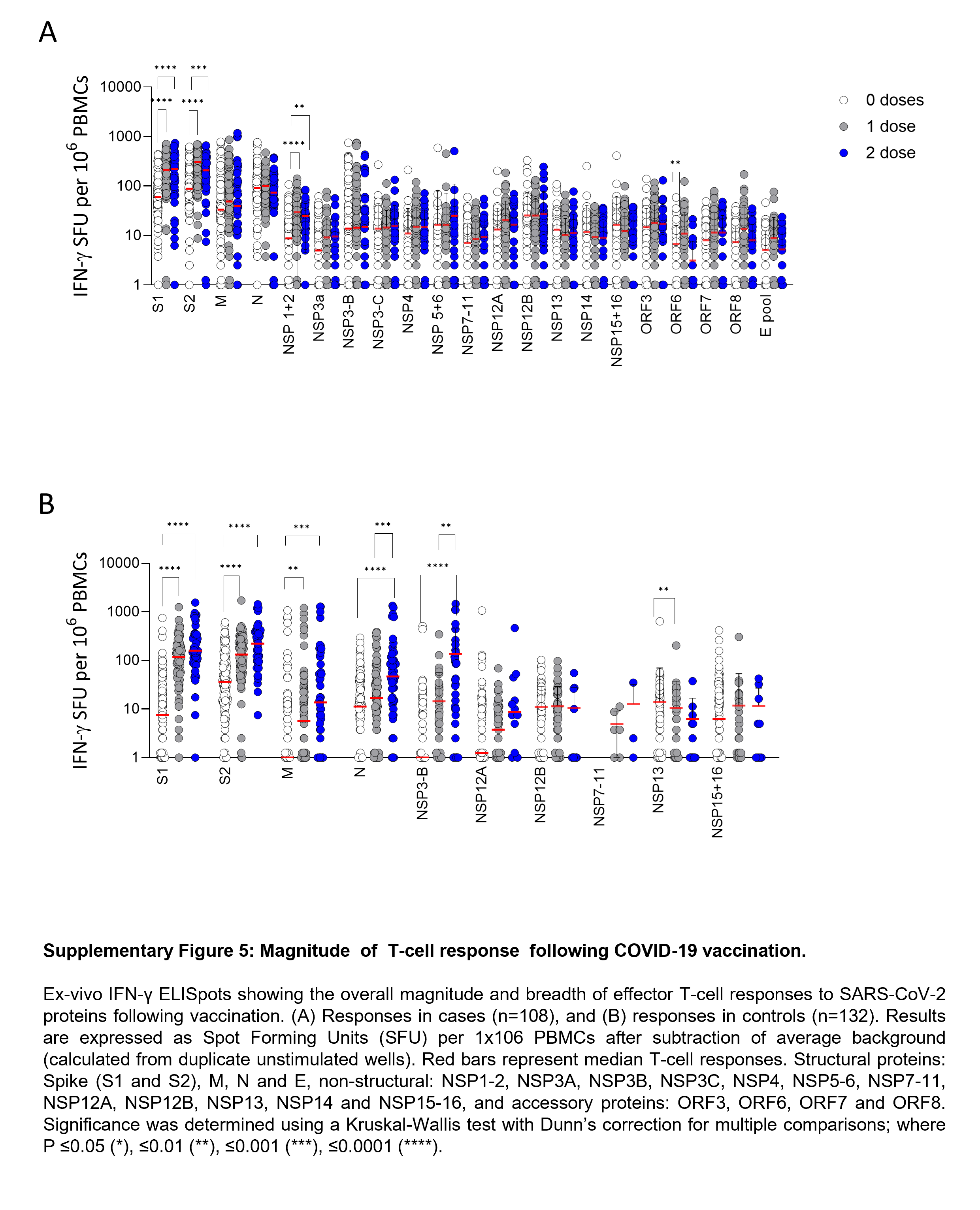

Supplement: jiaf246_Supplementary_Data [file jiaf246_supplementary_data.zip › Supplementary_Figure_5.png]

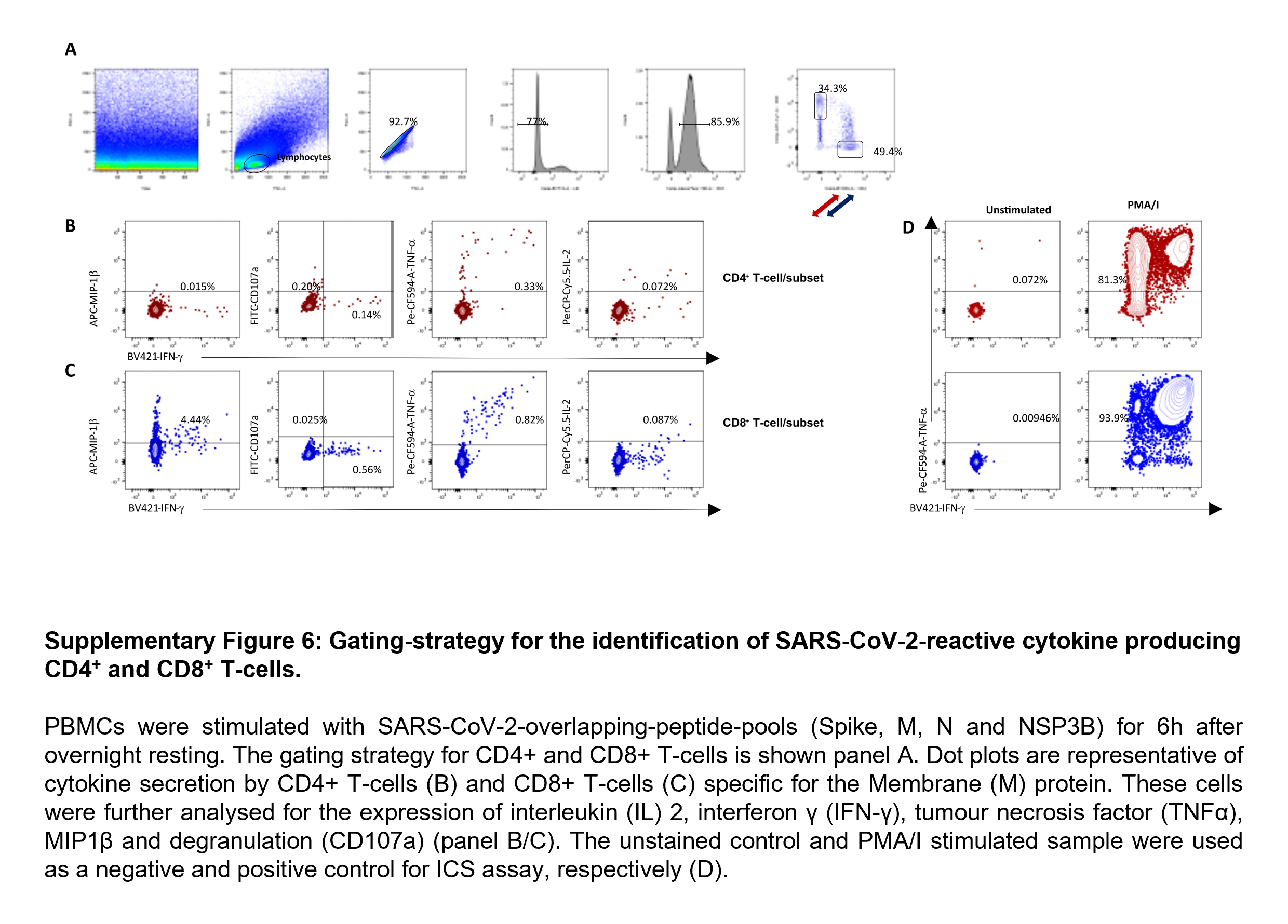

Supplement: jiaf246_Supplementary_Data [file jiaf246_supplementary_data.zip › Supplementary_Figure_6.png]

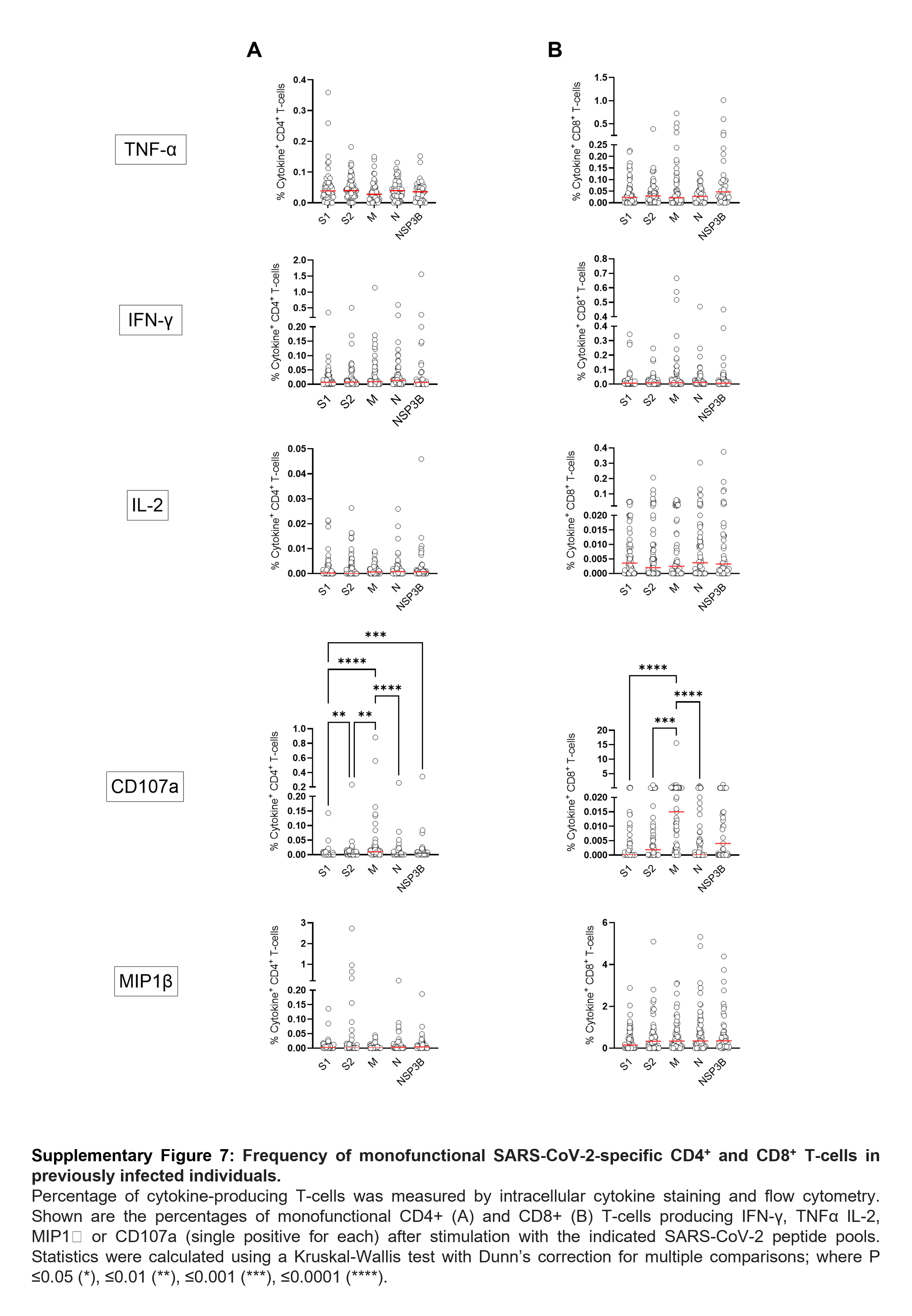

Supplement: jiaf246_Supplementary_Data [file jiaf246_supplementary_data.zip › Supplementary_Figure_7.png]

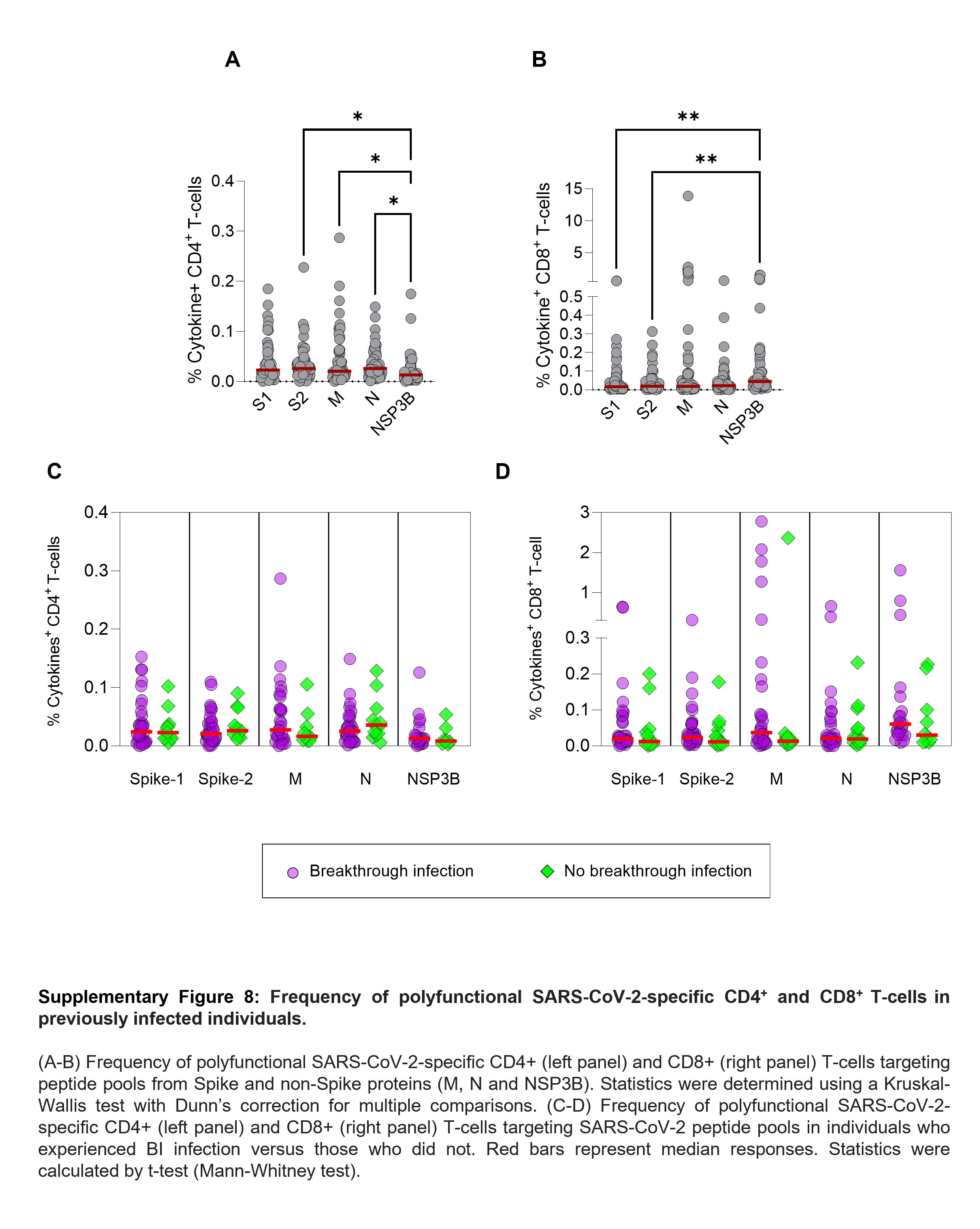

Supplement: jiaf246_Supplementary_Data [file jiaf246_supplementary_data.zip › Supplementary_Figure_8.png]

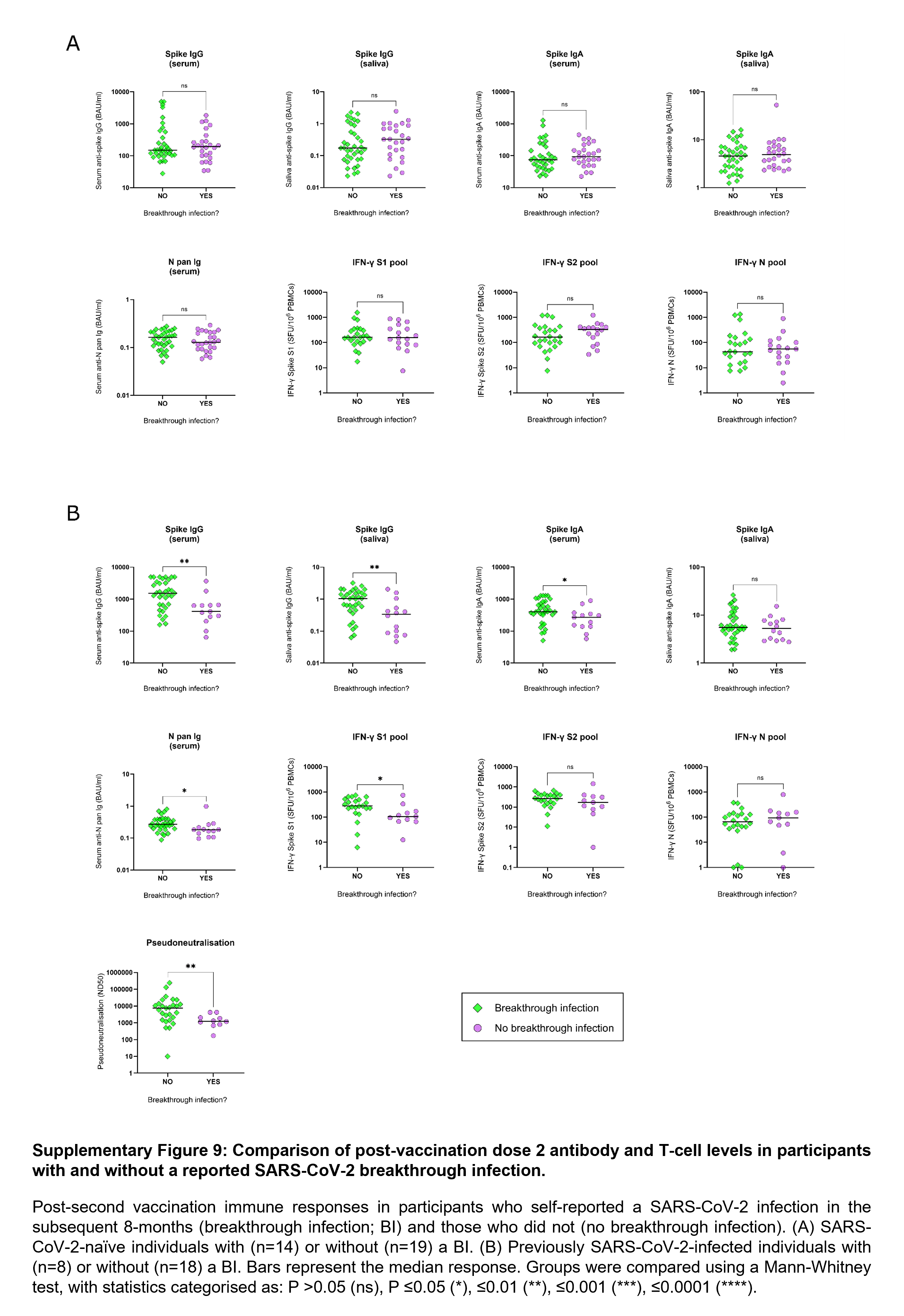

Supplement: jiaf246_Supplementary_Data [file jiaf246_supplementary_data.zip › Supplementary_Figure_9.png]

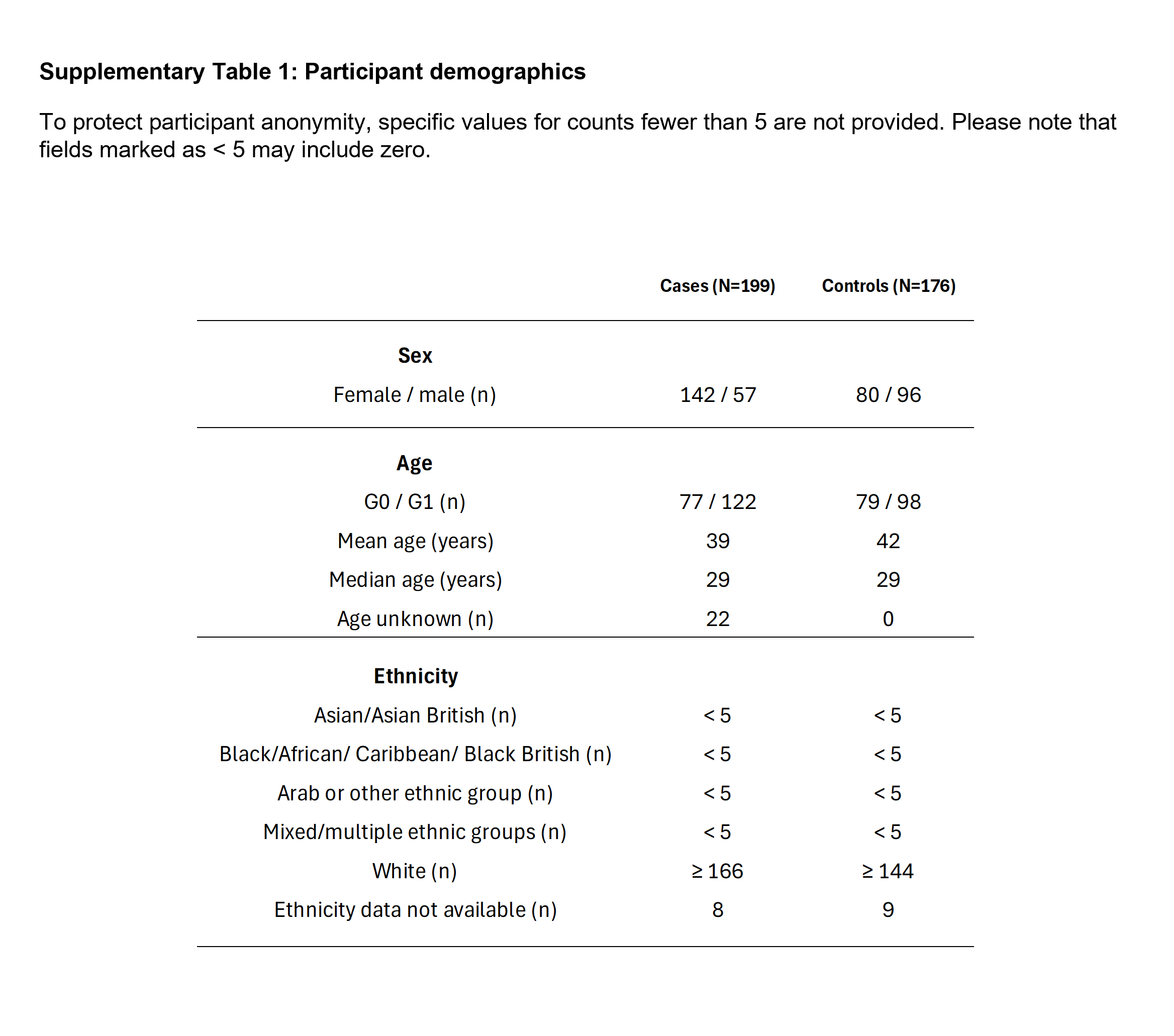

Supplement: jiaf246_Supplementary_Data [file jiaf246_supplementary_data.zip › Supplementary_Table_1.png]

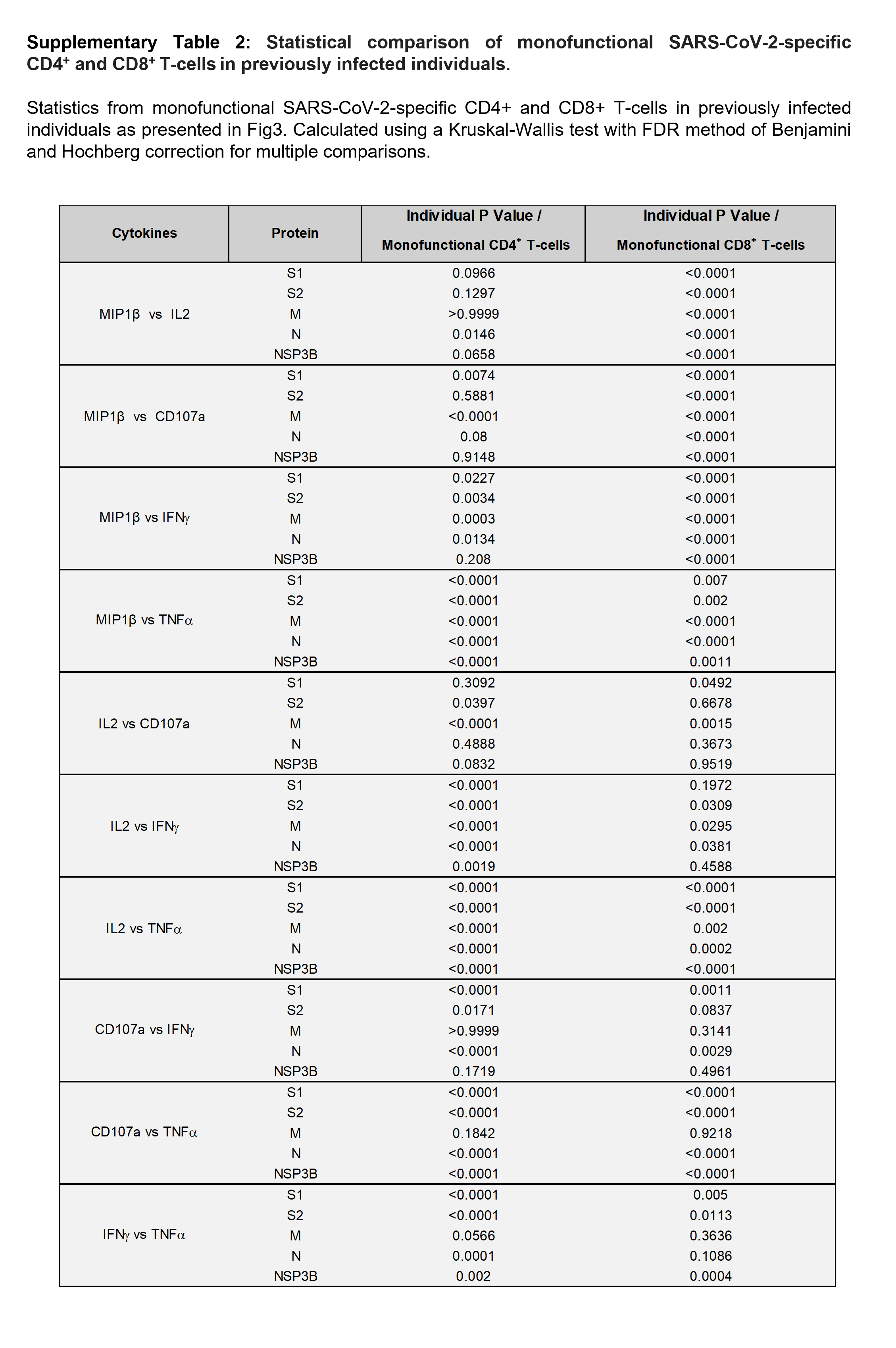

Supplement: jiaf246_Supplementary_Data [file jiaf246_supplementary_data.zip › Supplementary_Table_2.png]

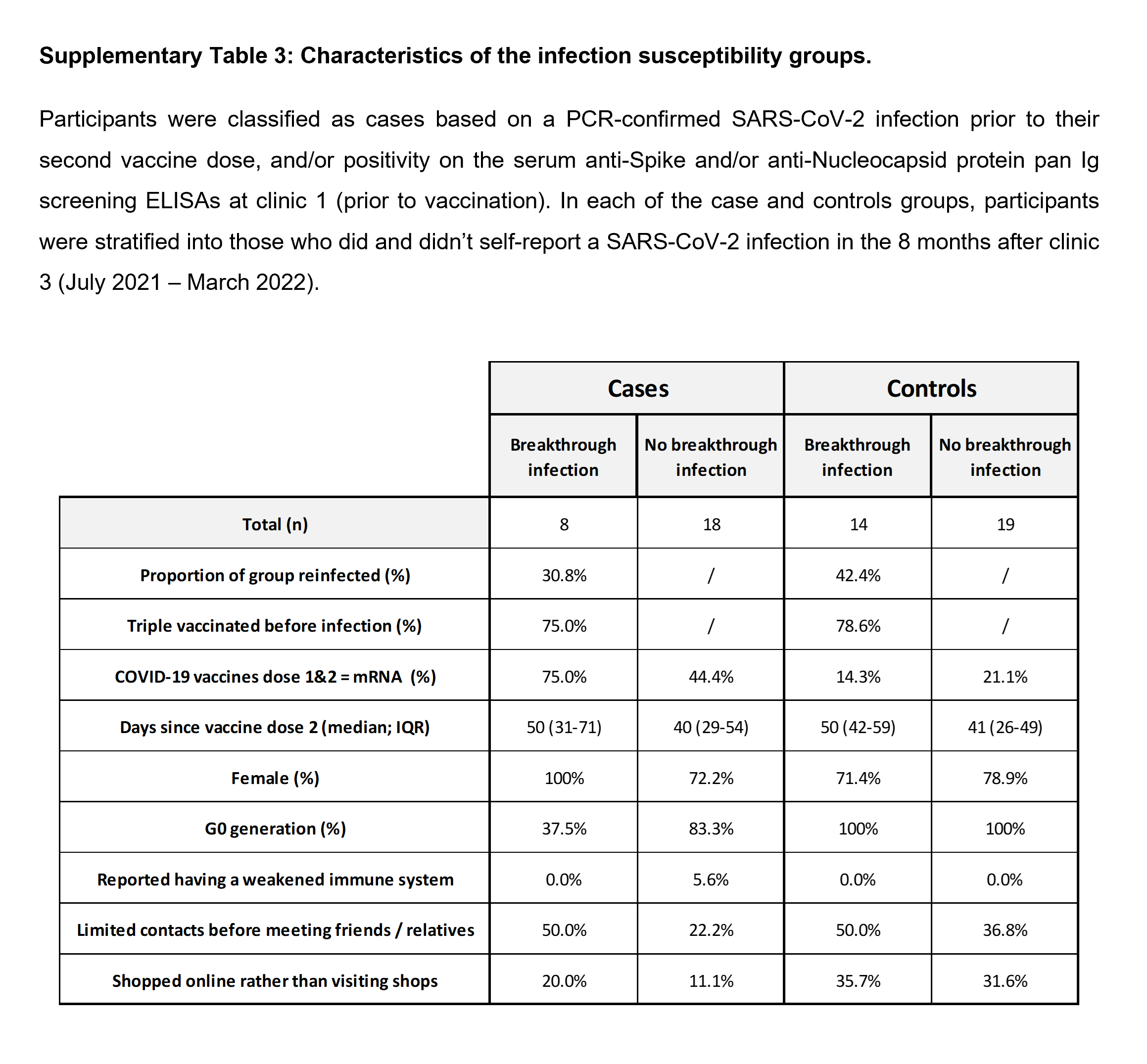

Supplement: jiaf246_Supplementary_Data [file jiaf246_supplementary_data.zip › Supplementary_Table_3.png]

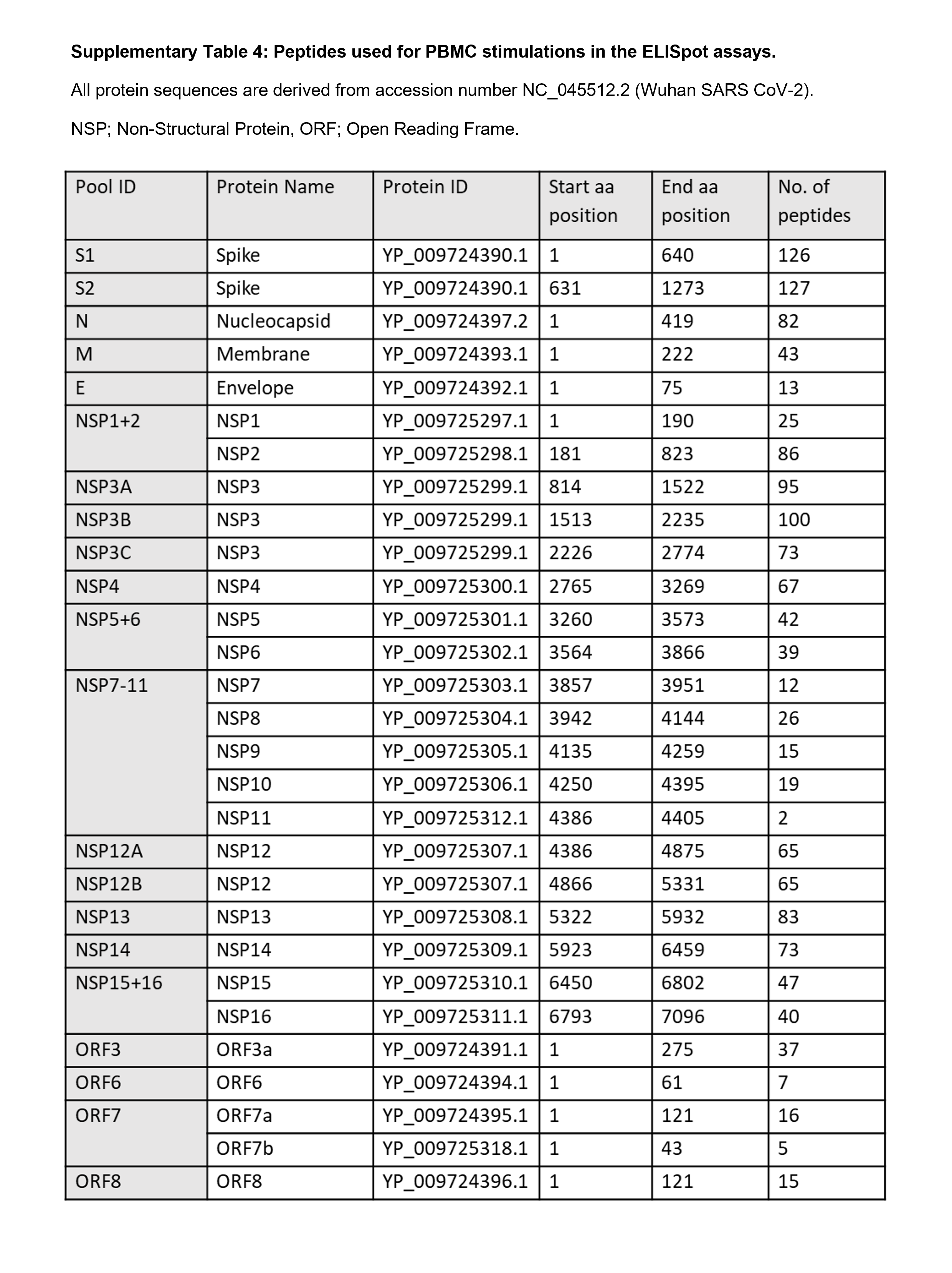

Supplement: jiaf246_Supplementary_Data [file jiaf246_supplementary_data.zip › Supplementary_Table_4.png]
